# Airway Secretory Cells Contain Both a Perinuclear Golgi Ribbon and Dispersed Golgi Satellites

**DOI:** 10.1093/ajrcmb/aanag018

**Published:** 2026-07-01

**Authors:** Oanh N. Hoang, Colin E. Chan, Joshua M. Brenner, Denisse Leza-Rincon, Ana M. Jaramillo, Brendan Dolan, Adam W. Aziz, Rodolfo A. Cardenas, Gerardo J. Cardenas, Eduardo D. Galvez, Joshua B. Hales, Emilia S. Nunez-Pena, Boxuan Yang, Reid T. Powell, Leoncio Vergara, Harry Karmouty-Quintana, Jesper M. Magnusson, Gunnar C. Hansson, Roberto Adachi, John D. Dickinson, Christopher M. Evans, Justin A. Courson, Alan R. Burns, Michael J. Tuvim, Burton F. Dickey

**Affiliations:** 1 Department of Pulmonary Medicine, The University of Texas MD Anderson Cancer Center, Houston, Texas, USA; 2 Division of Pulmonary Sciences and Critical Care Medicine, University of Colorado School of Medicine, Aurora, Colorado, USA; 3 Department of Medical Biochemistry and Cell Biology, University of Gothenburg, Gothenburg, Sweden; 4 Institute of Biosciences and Technology, Texas A&M School of Medicine, Houston, Texas, USA; 5 Department of Biochemistry and Molecular Biology and Division of Pulmonary, Critical Care, and Sleep Medicine, University of Texas Health Science Center at Houston, Houston, Texas, USA; 6 Department of Pulmonary Medicine, Institute of Medicine, University of Gothenburg, Gothenburg, Sweden; 7 Department of Internal Medicine, University of Nebraska Medical Center, Omaha, Nebraska, USA; 8 Research Services, Rocky Mountain Regional VA Medical Center, Aurora, CO, USA; 9 Department of Medicine, Baylor College of Medicine, Houston, Texas, USA; 10 Michael E. DeBakey Veterans Affairs Medical Center, Houston, TX, USA; 11 College of Optometry, University of Houston, Houston, Texas, USA.

**Keywords:** mucin, mucus, MUC5AC, MUC5B, Golgi

## Abstract

**Rationale:**

Finely tuned production and secretion of the polymeric mucins MUC5AC and MUCB are required for lung health, but knowledge of many details between their translation and their packaging into secretory granules is lacking.

**Objective:**

To analyze the structure and function of the Golgi apparatus, a key site of mucin glycosylation, folding, polymerization and packaging, in airway epithelial secretory cells.

**Methods:**

Lung tissue was obtained from mice stimulated or not with IL-13 to upregulate mucin production, and from normal human lungs. Golgi elements in mouse and human tissue were imaged by high-resolution immunofluorescence microscopy and electron microscopy. Tissue from mice with deletion of both polymeric mucins was also examined.

**Measurements and Main Results:**

By immunofluorescence microscopy, both mouse and human airway secretory cells contained ~100 dispersed puncta labeled by markers of medial and trans Golgi cisternae and the trans-Golgi network (TGN), but only a few perinuclear puncta were labeled by markers of cis-Golgi cisternae. By electron microscopy, secretory cells contained both a perinuclear Golgi ribbon and numerous dispersed Golgi stacks, termed satellites. In mucous metaplastic cells, satellites were concentrated among immature mucin granules. Increasing mucin production by cytokine stimulation did not increase the number of TGN puncta, nor did preventing polymeric mucin production by gene deletion reduce TGN puncta.

**Conclusions:**

Mucin-producing airway secretory cells express an unusual Golgi structure consisting of a conventional perinuclear ribbon as well as dispersed satellites. While the Golgi satellites are likely an adaptation for mucin production and packaging, their presence is specified developmentally, independent of mucin production.

## INTRODUCTION

The physical properties of airway mucus arise from interactions in the airway lumen of the secreted polymeric mucins, MUC5AC and MUC5B, with water and salts (1–3). These mucins are enormous glycoproteins, with sizes as monomers greater than two million Daltons, and they polymerize into linear chains containing tens of monomers. The huge size of mucin polymers is required for effective mucociliary clearance (4), but it places great demands on airway secretory cells that leads to proteostasis stress (5). Finely tuned production and secretion of MUC5AC and MUC5B are required for lung health. The absence of MUC5AC in mice results in impaired trapping of helminthic larvae migrating through the lungs (6), but hyperexpression of MUC5AC combined with rapid secretion in mice and humans causes mucus plugging of airways (7–11). The absence of MUC5B in mice and in humans results in impaired mucociliary clearance leading to airway inflammation, infection, and injury (12, 13). However, hyperexpression of MUC5B in mice increases susceptibility to bleomycin-induced lung injury (14, 15), and hyperexpression in humans is an important contributor to fibrotic interstitial lung diseases (16–19).

In view of the clinical importance of airway mucin production and secretion, the transcriptional control of MUC5AC and MUC5B synthesis and the mechanism of exocytic mucin secretion have been analyzed in detail (20–22). However, intermediate steps in mucin production, such as post-translational modifications including N-terminal polymerization and late stages of glycosylation, trafficking from endoplasmic reticulum through the Golgi apparatus, and packaging into secretory granules, have received less attention. Recently, we showed that most secretory granules contain both MUC5AC and MUC5B tightly interdigitating (23), similar to the packaging of two different mucins within single granules in *Drosophila* salivary glands (24). As our next goal in analyzing intermediate steps in mucin production, we sought to examine the packaging of mucins within granules together with exocytic proteins on the granule surface, such as VAMP-8 and Synaptotagmin-2 (25, 26), that make the granule competent for secretion. Co-packaging of mucins and exocytic proteins could be expected to occur in the trans-Golgi network (TGN), where assembly of secretory granules has been studied in multiple endocrine and exocrine cells (27, 28). However, when we visualized the TGN of airway secretory cells by immunofluorescence microscopy, we observed numerous widely dispersed puncta rather than concentrated perinuclear immunofluorescence as would be expected for a classical Golgi ribbon.

A Golgi apparatus is found in essentially all eukaryotic cells, but its appearance is highly variable across phyla. In vertebrates, the Golgi apparatus has a stereotypic structure consisting of several cisternal stacks linked laterally into a single ribbon (29–31). The ribbon is usually located just apical to the nucleus in polarized epithelial cells. While a ribbon is the most common Golgi structure in vertebrate cells, other structures can be observed in highly specialized cells. For example, Golgi mini-stacks termed satellites are seen in neuronal dendrites where they are thought to enable local protein synthesis without requiring lengthy transport from the cell soma (32–34). Another example is the occurrence of dispersed mini-stacks within endothelial cells where they are thought to enable the assembly of large von Willibrand factor polymers into secretory Weibel-Palade bodies (35–37). Besides its key roles in protein synthesis and trafficking, the Golgi apparatus is increasingly recognized for providing a platform for multiple cellular processes including cytoskeletal organization, sensing of proteostasis stress, metabolism, autophagy, inflammation, and apoptosis (29, 33). These activities can be modulated by changes in Golgi structure, so knowledge of the structure and function of the Golgi apparatus in lung parenchymal cells is important to understand lung pathophysiology. Here we determine the ultrastructure of Golgi stacks in airway epithelial secretory cells, their association with secretory mucins, and their developmental specification.

## METHODS

Briefly, wild-type C57BL/6 mice, challenged or not with cytokines to induce mucous metaplasia, were studied at MD Anderson Cancer Center under approved institutional protocols. MUC5AC/MUC5B double deletant mice were generated at the University of Colorado under approved institutional protocols. Normal human tissue was obtained at the University of Texas Health Science Center at Houston and the University of Gothenburg under approved institutional protocols. For microscopic analysis, widefield deconvolution immunofluorescence microscopy was performed at MD Anderson Cancer Center, electron microscopy at the University of Houston College of Optometry, and high resolution Airyscan immunofluorescence microscopy at the University of Gothenburg. Detailed methods are provided in the Online Supplement.

## RESULTS

### The trans-Golgi network (TGN) of mouse airway secretory cells is widely dispersed

As an initial step towards visualizing the assembly of mucin secretory granules, we imaged the TGN in airway epithelium by brightfield fluorescence microscopy using an antibody against TGN46. Rather than finding a few fluorescent puncta near the nucleus suggesting the Golgi ribbon of a typical mammalian cell (34), we observed numerous puncta widely distributed throughout the cytoplasm of secretory cells (not shown). In a pilot study to confirm the large number of TGN elements and their widespread distribution, we performed laser confocal immunofluorescence microscopy of naïve, uninflamed airway epithelial cells and cells with mucous metaplasia from IL-13 instillation (Figure E1). Using a volumetric algorithm to estimate the number of TGN elements per cell (Supplemental Methods), we found a mean of 87 puncta in naïve secretory cells versus 11 in ciliated cells, and 113 puncta in metaplastic secretory cells versus 11 in ciliated cells. Besides this numerical difference, TGN46 was distributed throughout the cytoplasm of secretory but not ciliated cells, extending close to the apical surface of tall metaplastic secretory cells distended with mucin granules (Figure E1B).

**Figure 1.**
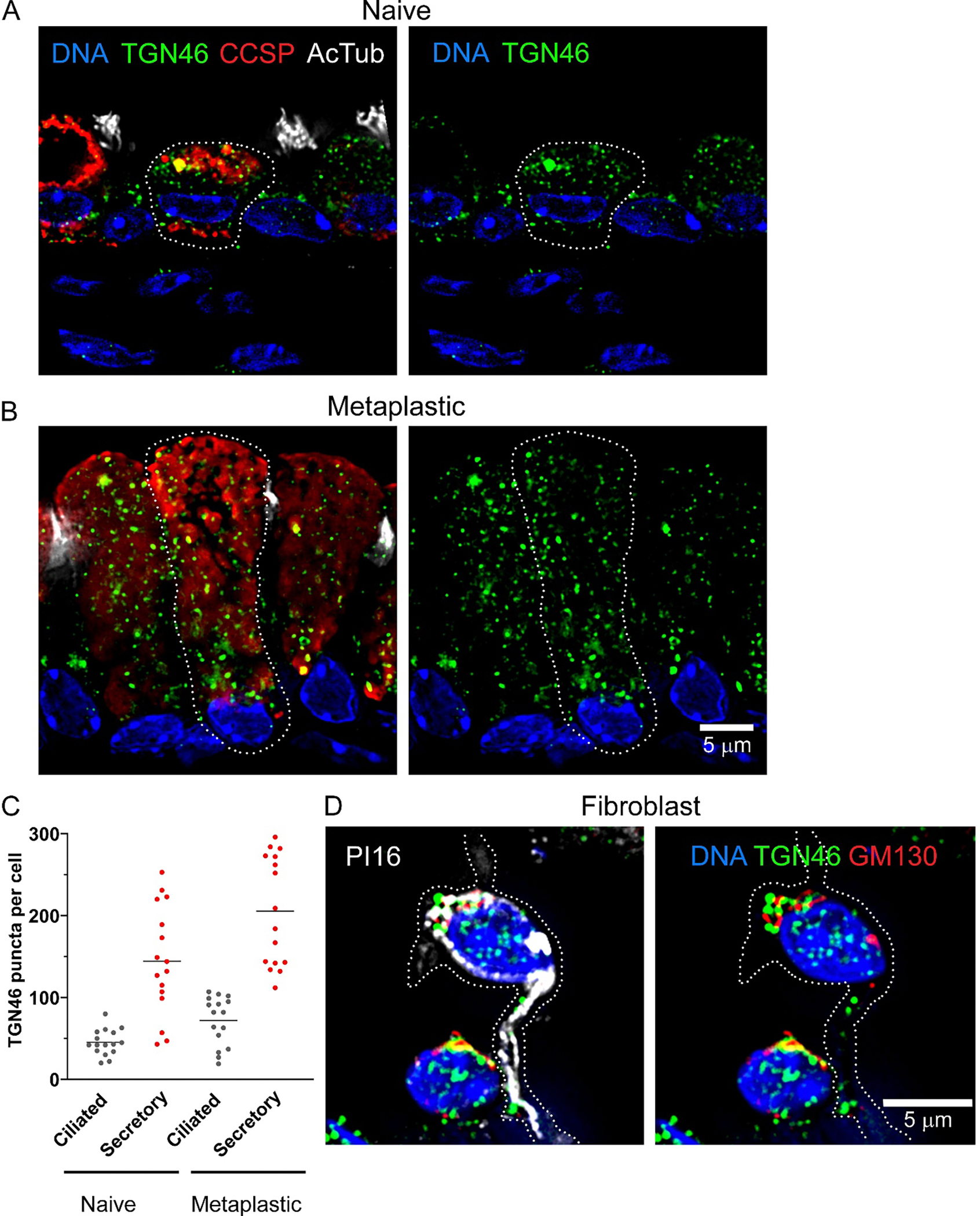
Numerous widely distributed trans-Golgi network (TGN) elements in mouse airway secretory cells. (*A)* Immunofluorescence deconvolution microscopy using antibodies against CCSP to mark secretory cells, acetylated tubulin (AcTub) to mark ciliated cells, TGN46 to mark the trans-Golgi network, and DAPI to stain nuclei in the axial bronchi of mice not exposed (naïve) to cytokines. The scale bar in *B* also applies here. (*B*) Microscopy as in *A*, but in mice with mucous metaplasia due to treatment with 1 μg IL-13. (*C*) TGN puncta were enumerated in 16 cells from 3 mice in each group. Selected comparisons were subjected to Mann-Whitney test with Bonferroni correction, which set significance at *p* < 0.0125. For naïve ciliated vs naïve secretory, *p* < 0.0001; for metaplastic ciliated vs metaplastic secretory, *p* < 0.0001; for naïve ciliated vs metaplastic ciliated, *p* = 0.01; for naïve secretory vs metaplastic secretory, *p* = 0.02. (D) Microscopy as in *A* of a submucosal fibroblast marked by the expression of PI16 (left panel), and showing TGN46 puncta aggregated with puncta of the cis-Golgi marker GM130 adjacent to a single pole of the nucleus (right panel).

To further assess these initial findings, we imaged mouse axial bronchi by widefield deconvolution immunofluorescence microscopy using a similar volumetric algorithm. This showed a mean of 144 TGN46 puncta in naïve secretory cells versus 45 in ciliated cells, and 206 puncta in metaplastic secretory cells versus 72 in ciliated cells (Figure 1). Again, TGN46 was widely distributed in secretory but not ciliated cells, extending to the apex of metaplastic secretory cells (Figures 1, E2). For comparison to a cell with a more conventional Golgi structure, we imaged submucosal fibroblasts marked by peptidase inhibitor 16 (PI16, Figure 1D). The fibroblasts showed TGN46 puncta adjacent to one side of the nucleus interspersed with GM130 puncta that mark the cis-Golgi, consistent with a Golgi ribbon containing complete stacks from cis to trans cisternae. Thus, mouse airway secretory cells show a larger number and wider dispersion of TGN elements than adjacent ciliated epithelial cells and submucosal fibroblasts.

### Mouse airway secretory cells contain both a conventional Golgi ribbon and dispersed Golgi satellites by electron microscopy (EM)

**Figure 2.**
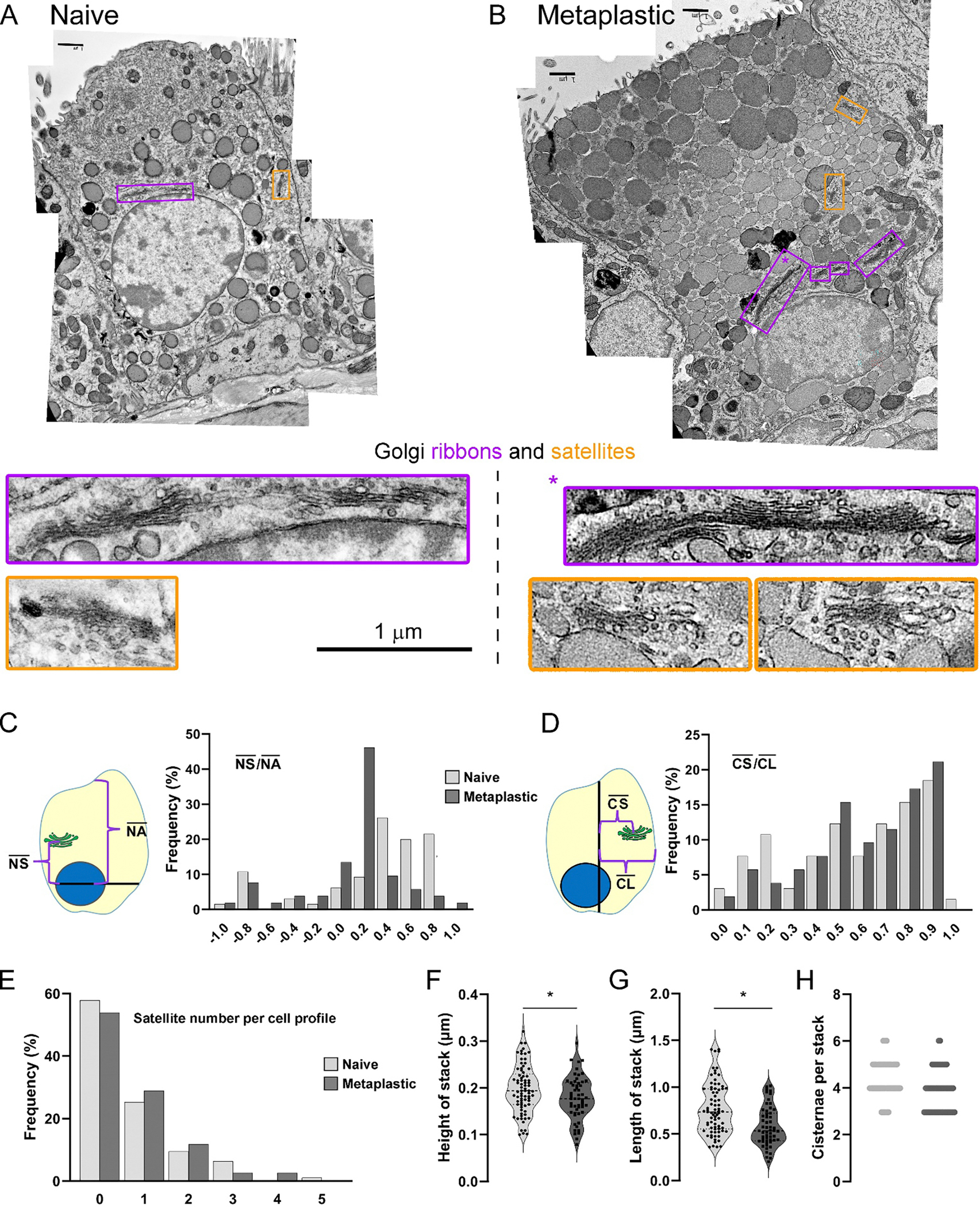
Electron microscopic analysis of Golgi structure in mouse airway secretory cells. Cellular profiles were compiled from multiple electron micrographs of secretory cells in mouse axial bronchi to include apical, basal, and lateral plasma membranes as well as a portion of the nucleus. Cellular profiles were compiled for 95 naïve (uninflamed) cells and 76 metaplastic cells. *(A)* Representative airway secretory cell of a naïve mouse. The Golgi ribbon is outlined in magenta, a Golgi satellite is outlined in orange, and both are also shown at higher magnification. All scale bars in *A* and *B* are 1 μm. *(B)* Representative metaplastic secretory cell in the airway of a mouse treated with 0.2 μg IL-13. The Golgi ribbon and satellites are outlined as in *A*, but only the portion of the ribbon indicated by an asterisk is shown at higher magnification. *(C)* Frequency distribution of the vertical position of satellites relative to the nucleus (NS) as a fraction of the distance from the center of the nucleus to the middle of the apical membrane (NA). Intervals extend 0.1 units more and less than the listed fractions, and positions below the center of the nucleus are negative. The vertical distribution of satellites differs between naïve and metaplastic cells, *p*<0.0001. *(D)* Frequency distribution of the lateral position of satellites relative to the center of the cell (CS) as a fraction of the distance from the center of the cell to the lateral membrane on that side at the level of the satellite (CL). Intervals extend 0.05 units more and less than the listed fractions. The horizontal distribution of satellites does not differ between naïve and metaplastic cells, *p* = 0.707. *(E)* Frequency distribution of the number of satellites per cellular profile shows no difference between naïve and metaplastic cells, *p* > 0.99. The morphology of satellites in naïve and metaplastic secretory cells was compared based on height *(F; p*=0.046*),* length *(G; p* < 0.01*)*, and number of cisternae per satellite*(H; p*<0.01). Statistical comparisons were done by the Kolmogorv-Smirnov test (*C-E*) and Mann-Whitney *U* test (*F-H*).

To characterize the structure of the Golgi apparatus in mouse airway secretory cells at higher resolution, we used transmission EM. Golgi stacks linked laterally in conventional ribbons were observed in both naïve and metaplastic secretory cells (Figure 2A–B). Most often these ribbons were located close to nuclei on the apical side. In addition to ribbons, isolated Golgi stacks were observed throughout the cytoplasm, most commonly apical or lateral to the nucleus rather than basal. We termed these scattered Golgi stacks “satellites” in reference to Golgi stacks found distant from ribbons in other cell types (34). In naïve secretory cells (Figure 2A), satellites were observed throughout the apical half of cells containing few mucin granules (note, atypical round mitochondria should not be mistaken for secretory granules, Figures E3, E4). In metaplastic secretory cells (Figure 2B), satellites were mostly observed among immature mucin granules in the middle third of cells; few satellites were observed among mature mucin granules in the apical third of cells or in the basal third that included the nucleus. Enlarged and annotated images of the cells in Figure 2A–B are provided in the Online Supplement, along with additional EM images (Figures E3, E4) and serial block-face scanning EM images (Figure E5; Videos 1, 2).

### Quantitative ultrastructural analysis of mouse Golgi satellites

Our qualitative observations were extended quantitatively by measuring the distribution of satellites relative to cell borders, and the number and dimensions of satellites in EM cellular profiles of 95 naïve cells and 76 metaplastic cells. Criteria for the identification of secretory cells and the compilation of EM images to generate cross-sectional profiles are provided in Supplemental Methods. In naïve cells, satellites concentrated at a fractional distance from the nucleus to the apical membrane of 0.4–0.8, whereas in metaplastic cells, they concentrated at a fractional distance of 0–0.4 (Figure 2C), consistent with the qualitative redistribution away from the cell apex described above. There was a gradient of slightly increasing satellite number towards the lateral borders of both naïve and metaplastic cells with no observable redistribution with metaplasia (Figure 2D).

The number of satellites visible in an EM cellular profile varied from 0 to 5, with no significant difference in the means of 0.68 in naïve cells and 0.71 in metaplastic cells (Figure 2E). Based upon our estimate of the volume fraction of an airway cell sampled in an EM cellular profile, we calculate a mean total satellite number of 55.5 per naïve cell and 57.9 per metaplastic cell (see Supplemental Results). The mean height of satellites in naïve cells was 0.20 μm and in metaplastic cells was 0.18 μm (Figure 2F), the mean length of satellites in naïve cells was 0.78 μm and in metaplastic cells was 0.58 μm (Figure 2G), and the mean number of cisternae in satellites in naïve cells was 4.4 and in metaplastic cells was 3.9 (Figure 2H). Thus, while there is a substantial redistribution of satellites during mucous metaplasia towards the middle third of secretory cells where immature mucin granules are concentrated, there was no significant difference between naïve and metaplastic cells in the number of satellites and only small differences in satellite dimensions.

### Cis-Golgi cisternae are concentrated in the ribbon whereas trans-Golgi cisternae are present in both the ribbon and satellites in mice

**Figure 3.**
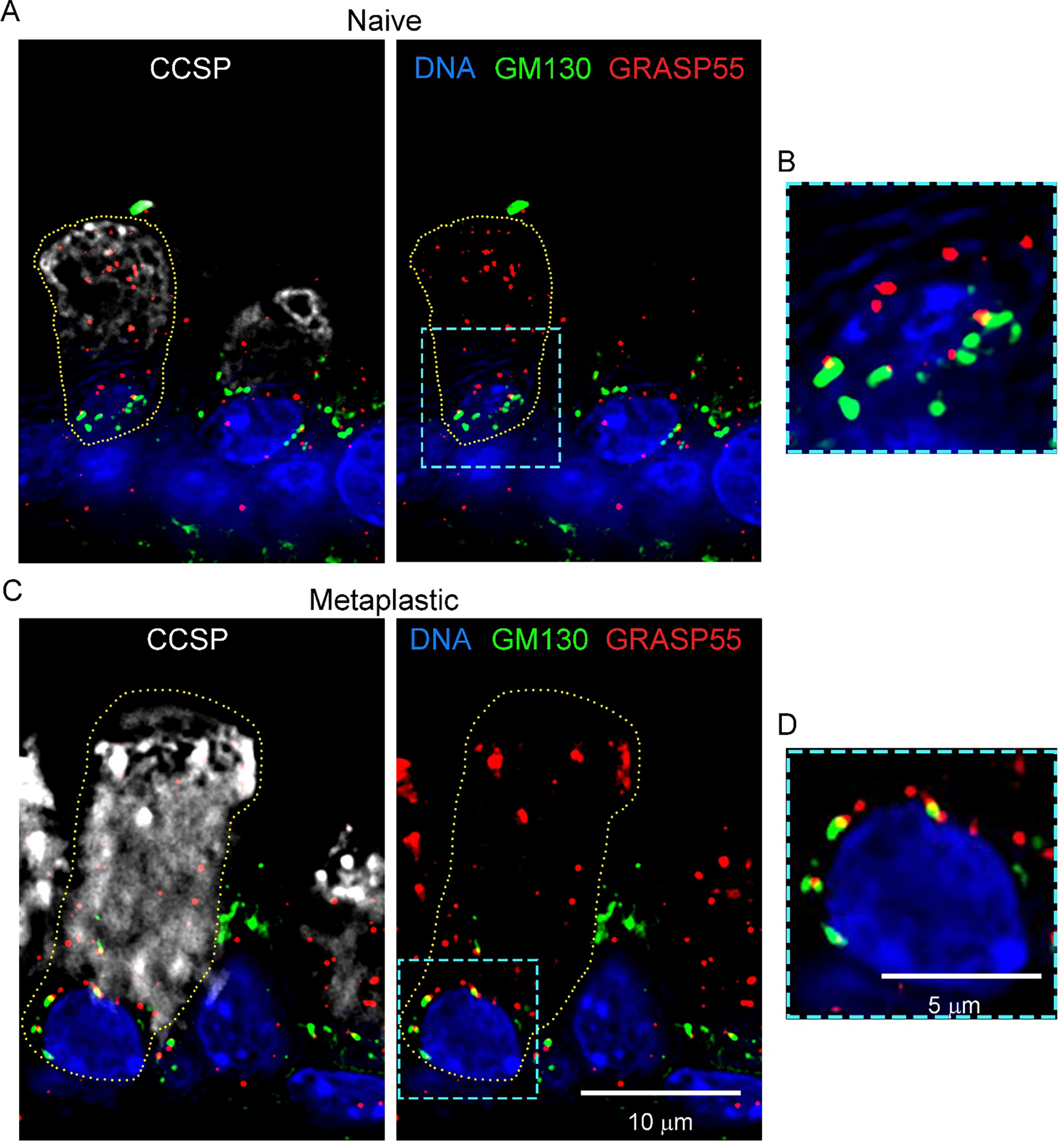
Localization of cis-Golgi and trans-Golgi cisternae in mouse airway secretory cells. *(A)* Immunofluorescence deconvolution microscopy using antibodies against CCSP to mark secretory cells, GM130 to mark cis-Golgi cisternae, GRASP55 to mark trans-Golgi cisternae, and DAPI stain to mark nuclei in the axial bronchus of naïve mice. *(B)* The perinuclear area outlined in *A* is shown at higher magnification. *(C)* Microscopy as in *A*, but in mice with mucous metaplasia due to treatment with 1 μg IL-13. *(D)* The perinuclear area outlined in *C* is shown at higher magnification.

To determine whether Golgi stacks in the ribbon and satellites had the same or a different composition, we performed immunofluorescence deconvolution microscopy using antibodies against the cis-Golgi marker GM130 and the trans-Golgi marker GRASP55. GM130 was concentrated in the perinuclear region of both naïve and metaplastic secretory cells, similar to its distribution in neighboring ciliated cells and consistent with predominant localization in the Golgi ribbon (Figures 3, E6). In contrast, GRASP55 was observed throughout the cytoplasm of secretory cells, consistent with localization in both the ribbon and satellites. GM130 and GRASP55 colocalized in the perinuclear region, consistent with their presence within a single Golgi stack in ribbons (yellow color in Figures 3, E6). To observe the localization of GM130 and TGN46 in relation to mucin granules, antibodies to MUC5B and MUC5AC were used. GM130 was seen in a perinuclear location not overlapping MUC5B in either naïve or metaplastic cells (Figure E7A–B). In contrast, TGN46 was seen throughout the cytoplasm, including in the region of MUC5B granules in naïve and metaplastic cells (Figure E7C–D), and the region of MUC5AC in metaplastic cells (Figure E7F).

### TGN and trans-Golgi cisternae in human airway secretory cells are widely dispersed

**Figure 4.**
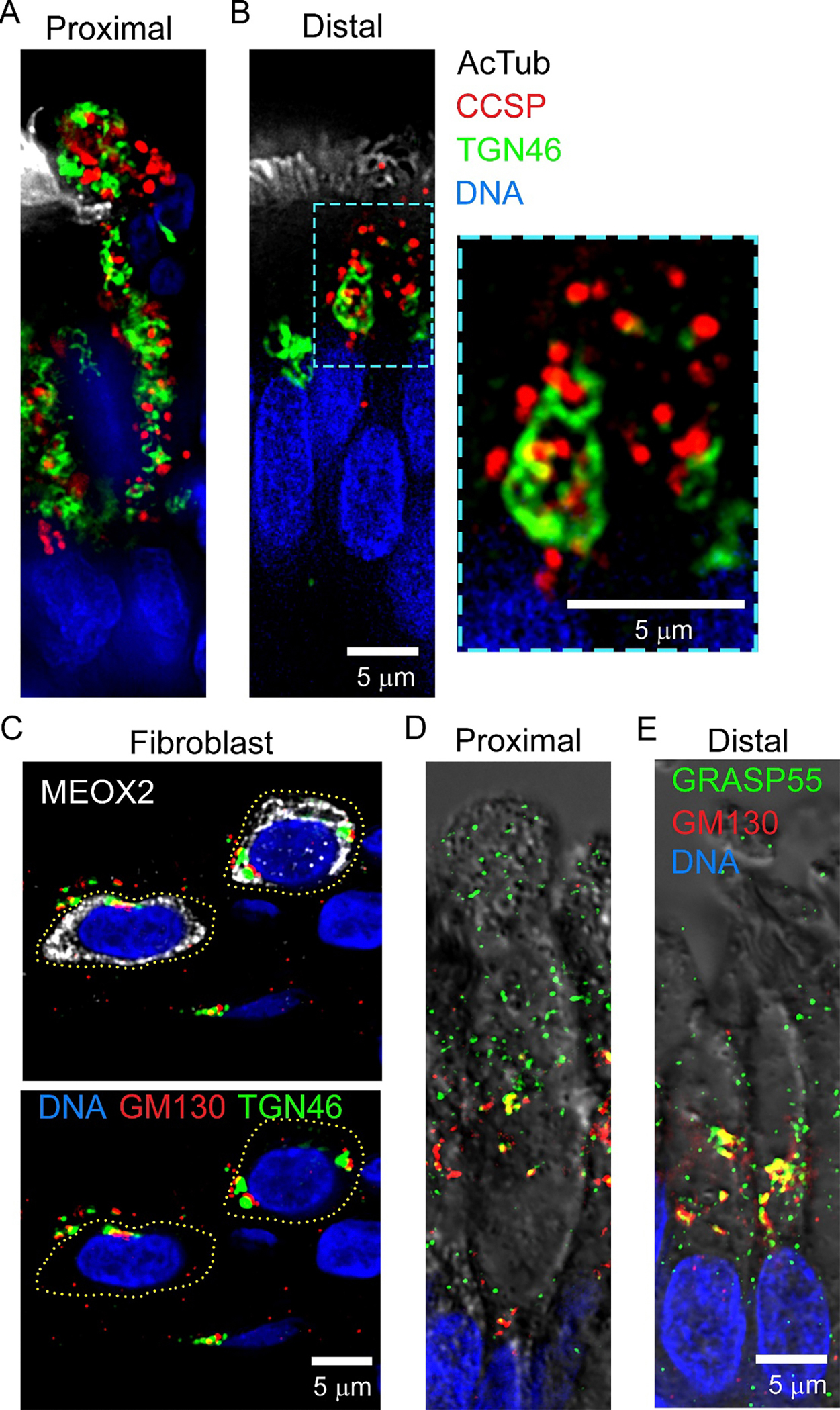
Localization of Golgi cisternae in human airway secretory cells. *(A)* Immunofluorescence deconvolution microscopy using antibodies against acetylated tubulin (AcTub) to mark ciliated cells, CCSP to mark secretory cells, TGN46 to mark the trans-Golgi network, and DAPI to mark nuclei in a proximal human airway. *(B)* Microscopy as in *A*, but in a distal human airway. The area marked in the secretory cell is shown at higher magnification on the right. *(C)* Microscopy as in *A*, but using antibodies against MEOX2 to mark fibroblasts and antibodies against GM130 to mark cis-Golgi cisternae. The MEOX2 channel is left out of the bottom image to better visualize GM130 and TGN46. *(D-E)* Microscopy as in *A-B*, but using antibodies against GRASP55 to mark trans-Golgi cisternae and against GM130 to mark cis-Golgi cisternae. Fluorescence images are superimposed on differential interference contrast images.

To determine whether the structure of the Golgi apparatus in human airway secretory cells is similar to that in mice, we probed sections of proximal and distal human airways with antibodies against TGN46, GRASP55, and GM130 using deconvolution immunofluorescence microscopy. In proximal airways, antibodies against TGN46 showed widely dispersed TGN from the nucleus all the way to the apical plasma membrane of tall secretory cells (Figure 4A). Neighboring ciliated cells did not show similarly dispersed TGN46 staining. In distal airways, epithelial cells were shorter, but TGN46 staining again extended apically from the nucleus (Figure 4B). In both proximal and distal secretory cells, TGN46 morphology was both punctate and tubular, with many tubules apparently wrapped around CCSP-containing secretory granules (Figure 4B inset) and around mucin granules containing MUC5AC or MUC5B or both mucins (Figure E8). In contrast to the dispersed expression of TGN46 in secretory cells, submucosal fibroblasts identified by MEOX2 staining showed TGN46 in just one or two perinuclear puncta associated with the cis-Golgi marker GM130, consistent with their exclusive localization to a ribbon (Figure 4C).

To determine whether there is a spatial dissociation between cis and trans Golgi elements in human secretory cells as in mice, we used antibodies against GRASP55 that localizes to the trans-Golgi and against GM130 that localizes to the cis-Golgi. In proximal secretory cells, GRASP55 was widely dispersed throughout the cytoplasm, similar to the distribution of TGN46 (Figure 4D). In the shorter distal airway secretory cells, GRASP55 was also observed in both dispersed and perinuclear locations, though the dispersed element was less abundant than in proximal cells (Figure 4E). In contrast, GM130 was concentrated near the nucleus in both proximal and distal airways (Figure 4D–E), often colocalized with GRASP55 consistent with their joint presence in a Golgi ribbon (yellow color). Thus, similar to mice, human airway secretory cells show TGN and trans-Golgi elements widely dispersed, consistent with their presence in both satellites and the ribbon, whereas cis-Golgi elements are concentrated near the nucleus, consistent with their presence mostly in the ribbon.

### Human airway secretory cells contain dispersed Golgi satellites by EM

**Figure 5.**
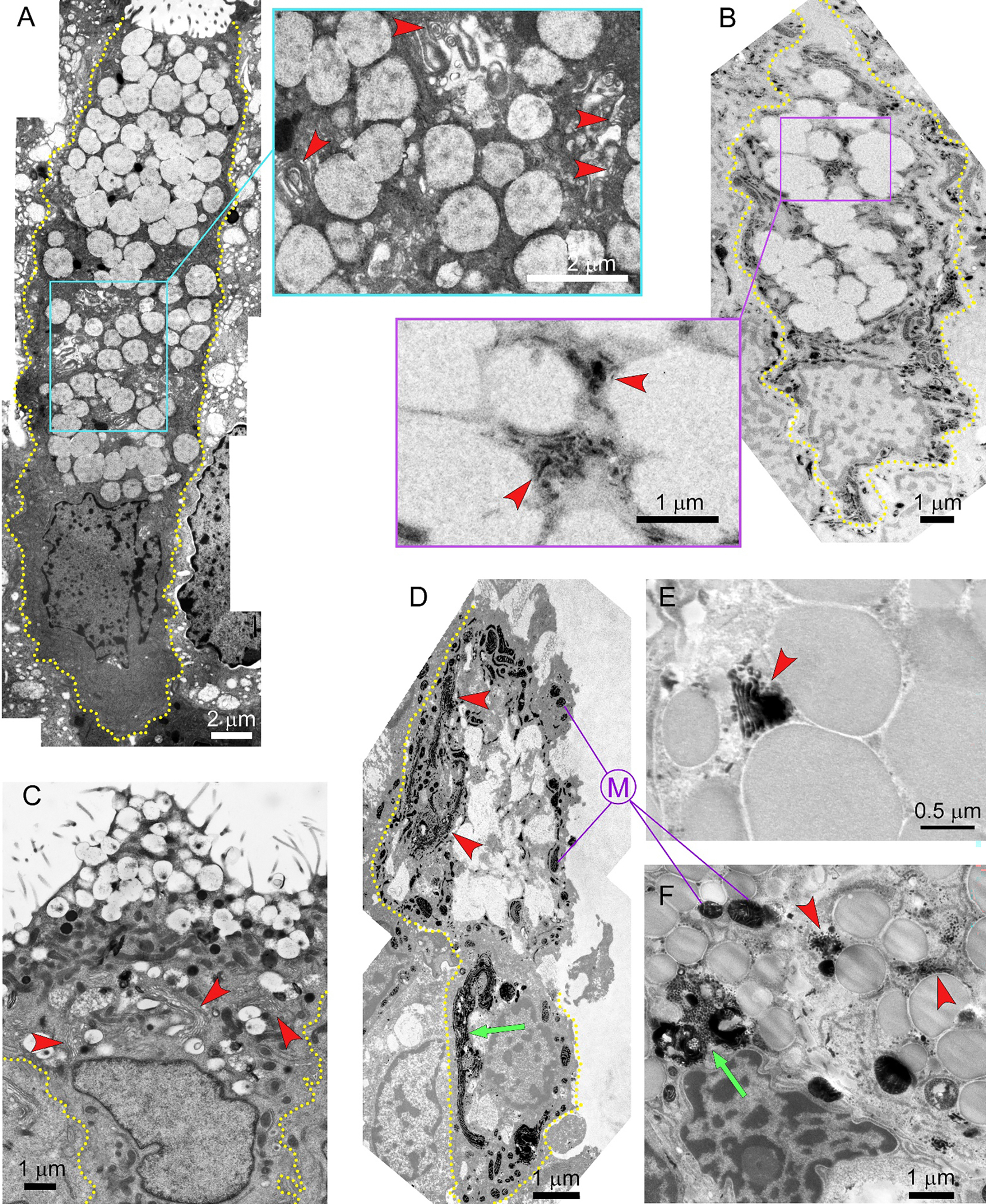
Electron microscopic images of Golgi structure in human airway secretory cells. *(A*) Profile of a human airway secretory cell compiled from multiple EM images of tissue fixed with cacodylate-buffered glutaraldehyde followed by cacodylate-buffered osmium tetroxide. The inset at high magnification shows multiple Golgi cisternae (red arrowheads) in close proximity to mucin granules. *(B)* Profile of a human airway secretory cell from EM images of tissue fixed and stained with zinc iodide – osmium tetroxide (ZIO) to highlight Golgi elements (red arrowheads) in close proximity to mucin granules. *(C)* EM profile of a cultured human airway epithelial secretory cell fixed with paraformaldehyde and glutaraldehyde, with Golgi cisternae (red arrowheads) seen both near the nucleus and in proximity to granules. *(D)* Partial EM profile of a cultured human airway epithelial secretory cell fixed and stained with ZIO, highlighting a perinuclear Golgi ribbon (green arrow) and peripheral Golgi satellites (red arrowheads), as well as stained mitochondria (purple M). *(E and F)* Golgi stacks (red arrowheads) adjacent to mucin granules in human submucosal gland mucous cells in tissue fixed and stained with ZIO, as well as a probable Golgi ribbon adjacent to a nucleus (green arrow in F).

To confirm that dispersed trans-Golgi elements observed by immunofluorescence microscopy of human airway secretory cells represent Golgi mini-stacks similar to those in mice, we performed transmission EM of human airway tissue and cultured human airway epithelial cells. In tissue sections, proximal airway secretory cells showed Golgi mini-stacks in close apposition to mucin granules in the middle third of cells (Figure 5A). Zinc iodide – osmium tetroxide (ZIO) fixation/staining confirmed the abundance of dispersed Golgi elements in mucin-containing secretory cells (Figure 5B). In cultured human airway epithelial cells, dispersed Golgi elements were observed both in unstained (Figure 5C) and ZIO-stained (Figure 5D) specimens, and perinuclear Golgi ribbons were also seen (Figure 5D). Golgi mini-stacks were also observed in close apposition to mucin granules in stained specimens of submucosal gland mucous cells (Figures 5E–F, E9).

### Golgi satellites are associated with immature mucin granules in human airway secretory cells

**Figure 6.**
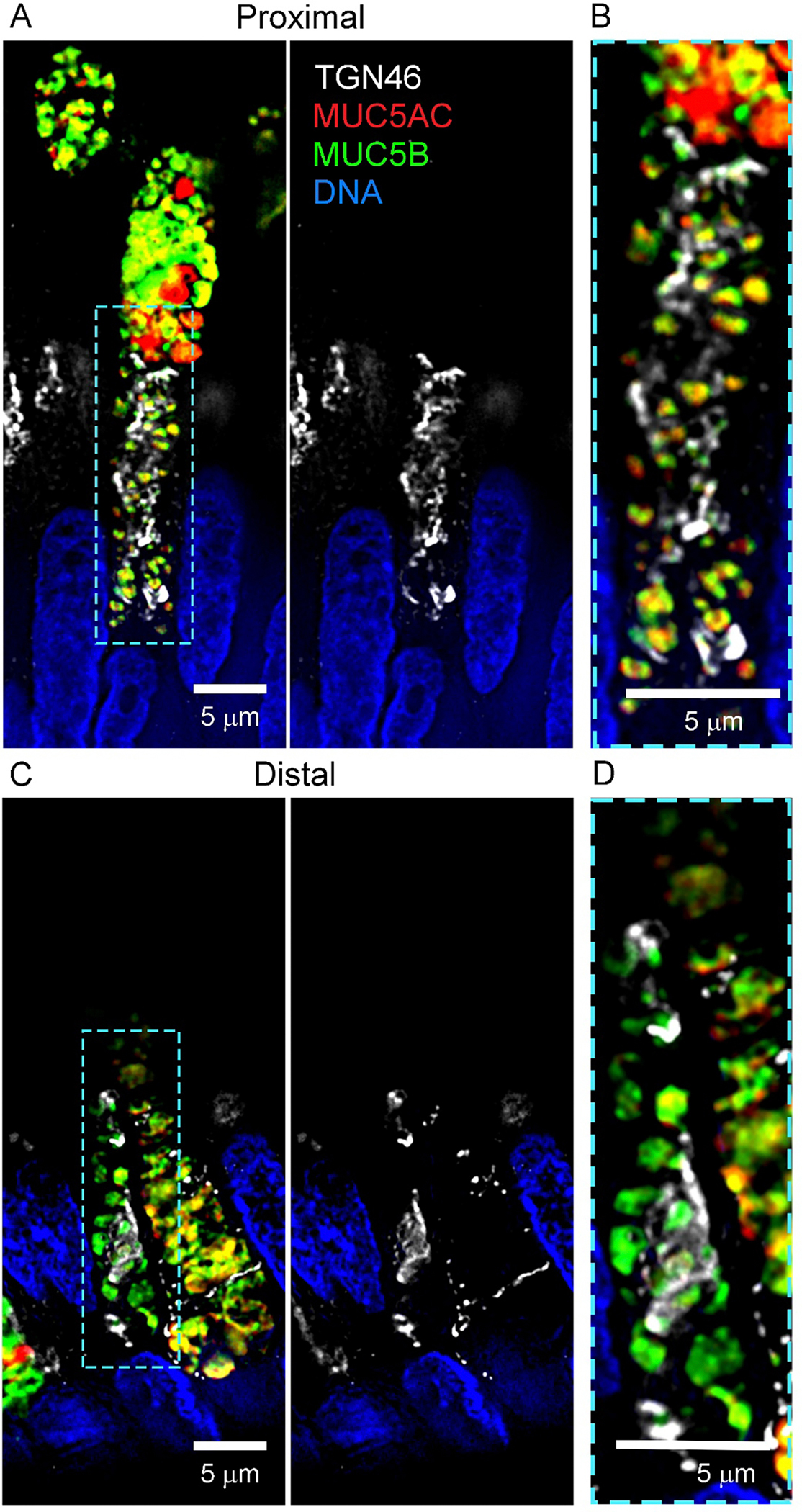
Localization of TGN in relation to mucin granules in human airway secretory cells. *(A)* Immunofluorescence deconvolution microscopy using antibodies against TGN46 to mark the trans-Golgi network, MUC5AC and MUC5B to mark mucin granules, and DAPI to mark nuclei in a proximal human airway. The MUC5AC and MUC5B channels are left out of the right image to better visualize TGN46. *(B)* The area marked in the middle of the secretory cell in *A* is shown at higher magnification. *(C)* Microscopy as in *A*, but in a distal human airway. *(D)* The area marked in the middle of the secretory cell in *C* is shown at higher magnification.

To determine whether Golgi satellites in human airway secretory cells are concentrated around immature mucin granules as in mice (Figures 1, 2), we localized TGN46 with MUC5AC and MUC5B by immunofluorescence deconvolution microscopy. In proximal airways, tall secretory cells often contained large granules staining for MUC5AC, MUC5B, or both mucins together in the apical third of their cytoplasm (Figure 6A). TGN46 was almost entirely excluded from this apical region containing mature mucin granules. In contrast, the middle third of these cells contained smaller, immature mucin granules with abundant TGN46 staining in puncta and tubules adjacent to the granules (Figure 6A–B, E8). In distal airways, secretory cells contained more MUC5B than MUC5AC, and TGN46 staining extended to the apical plasma membrane (Figure 6C–D). Distal secretory cells contained less MUC5AC than proximal cells, consistent with previous observations (23, 38), and the distribution of TGN46 throughout the cytoplasm of distal cells in close proximity to MUC5B granules suggests that these cells do not retain mature mucin granules but instead continuously secrete them as previously suggested (20, 39).

### Satellite TGN are a site of mucin packaging into granules

The concentration of Golgi satellites around immature mucin granules suggests that the presence of satellites in airway secretory cells is an adaptation for the synthesis and packaging of mucins. To address this, we examined normal human tracheal tissue for the association of incompletely glycosylated mucin proteins (iMUC5AC and iMUC5B) with granules, ER, and satellites. This was done using monoclonal antibodies raised against a non-glycosylated peptide from MUC5AC (40) and almost completely deglycosylated purified MUC5B (41), neither of which reacts against fully *O*-glycosylated mature mucins. To label mature mucin granules, we used monoclonal antibodies that react with fully glycosylated and folded MUC5AC (42) or MUC5B (43) (fMUC5AC and fMUC5B). Mature mucin granules near the apex of secretory cells, identified by the presence of fMUC5AC or fMUC5B (Figure E10A–B), did not show any reactivity with the antibodies against iMUC5AC and iMUC5B, which labeled small puncta in the middle third of cells. Some of the iMUC5AC and iMUC5B reactivity in smaller puncta appeared to overlap with the ER marker calnexin (yellow color, Figure E10C–D), and with nearby mucin granules appearing as black circles in those images. TGN46 immunofluorescence was observed adjacent to but not overlapping with puncta of iMUC5AC and iMUC5B (Figure E10E–F), suggesting that mucin glycosylation and folding are completed in adjacent satellite cisternae, and that the fully glycosylated mucins are then inserted into maturing granules at the TGN.

### Expression of Golgi satellites is upstream of mucin biosynthesis

**Figure 7.**
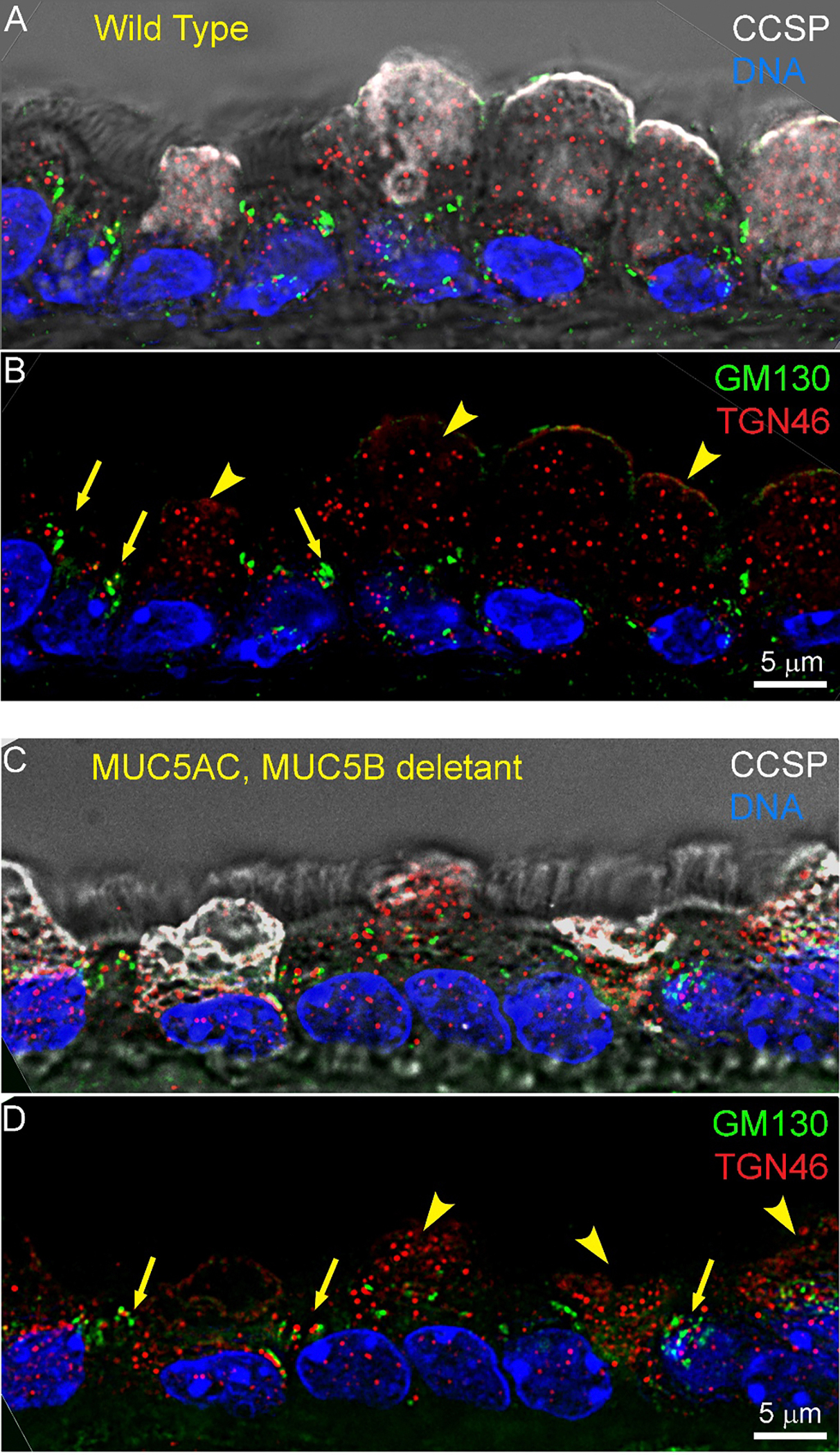
Distribution of Golgi cisternae in mouse airway secretory cells lacking polymeric mucins. *(A)* Merged deconvolution immunofluorescence and differential interference contrast (DIC) images of the axial bronchus of a naïve wild-type mouse without mucous metaplasia. Antibodies against CCSP mark secretory cells, GM130 marks cis Golgi cisternae, TGN46 marks the trans-Golgi network, and DAPI marks nuclei. (*B*) The same field as in *A* but without CCSP staining or the DIC image to better show staining of Golgi cisternae. GM130-labeled cis Golgi cisternae localize near the nucleus in both secretory and ciliated cells, whereas the TGN46-labeled trans-Golgi network localizes alongside GM130 puncta in ciliated cells (yellow arrows) but is dispersed throughout the cytoplasm in secretory cells (yellow arrowheads). *(C)* Microscopy is as in *A*, but in MUC5AC and MUC5B double deletant mice. *(D)* The same field as in *C* but without CCSP staining or the DIC image. As in wild-type mice, TGN46 is dispersed throughout the cytoplasm only in secretory cells.

Even though our findings suggest that the requirements of polymeric mucin synthesis and packaging drive the expression of Golgi satellites in airway secretory cells, the fact that the number of satellites changes minimally or not at all with mucous metaplasia (Figures E1, 1C, 2E) makes it unlikely that the synthesis of mucins itself drives satellite expression. To further address this, we examined secretory cells of mice in which both secreted polymeric mucins were deleted (Fig. E11). The epithelium of double knockout mice appeared shorter than that of wild-type mice (Figure 7A–B), consistent with prior observations that airway epithelial height depends in part on mucin production (Figures 1, 2, E1, E2, E6, E7). However, there was no apparent reduction in the abundance of dispersed TGN46 puncta in secretory cells of double knockout mice (Figure 7A–B).

## DISCUSSION

Here we analyze the structure of the Golgi apparatus as a key intermediate in mucin production and secretion. We find that airway secretory cells express an unusual Golgi structure consisting of both a conventional perinuclear ribbon and numerous unconventional satellites. Isolated Golgi stacks have occasionally been noted among mucin granules (44, 45), but no systematic characterization was performed. We find that satellites are de-enriched for a cis-Golgi marker relative to trans-Golgi and TGN markers. During mucous metaplasia, satellites redistribute from dispersion throughout the cytoplasm to concentration among immature mucin granules in the cell center. However, satellite number does not change with mucous metaplasia or deletion of polymeric mucin genes. These findings warrant further discussion.

The presence of dispersed Golgi stacks in specialized vertebrate cell types is hypothesized to be driven by various cell biological needs. These include local protein synthesis in axons and dendrites to avoid the need for long distance transport in neurons; serving as microtubule organizing centers for alignment of myofibrils in skeletal muscle; and the insertion of large secretory products into exocytic granules as for von Willebrand factor (VWF) in endothelial cells (29–37). The most extensively studied of these systems is the neuron, where two types of dispersed Golgi stacks have been identified – outposts and satellites. Outposts occur proximally in dendrites where they function both in localized secretion and in microtubule nucleation for dendritic branching (34, 46). Satellites occur distally in dendrites where they function in localized secretion of selected glycoproteins, are smaller than outposts, and lack the cis-Golgi structural protein GM130. In view of the wide dispersion of Golgi stacks in airway secretory cells, their association with immature mucin granules (Figures 2, 5, 6, E7–8), their small size (average 0.19 × 0.68 μm, 4 cisternae; Figure 2), and their depletion of GM130 (Figures 3, 4, E6), we think it most appropriate to designate them as satellites. It is likely that the very large size of secretory mucin polymers has driven the adaptation of Golgi satellite expression in airway secretory cells, similar to the situation with VWF polymers that share homology with mucins (35–37). This is supported by the redistribution of satellites towards close apposition to immature mucin granules in metaplastic cells (Figures 2, 5, 6, E8). By positioning satellites adjacent to nascent granules, mucin polymers could be directly transferred through tubules rather than requiring vesicular transport from a perinuclear ribbon, though this is speculative and would need to be established experimentally. It is also possible that the advantage of concentrating specialized glycosyltransferases and chaperones required for mucin synthesis in a distinct Golgi compartment drove the adaptation of satellite formation.

While the need to synthesize and package polymeric mucins likely drove the expression of satellites in airway secretory cells during mammalian evolution, it is striking that the number (Figures 1, 2, E1) and size (Figure 2F–H) of satellites does not rise substantially with increased mucin synthesis. The small increases in satellite number observed by immunofluorescence microscopy could be due to cellular expansion during mucous metaplasia allowing better visualization of fluorescent puncta (Figures 1, E1), which is supported by the lack of increase observed by EM (Figure 2E). The minor decreases in satellite dimensions during mucous metaplasia observed by EM are unlikely to be biologically significant in view of the small effect sizes and large confidence intervals (Figure 2F–H), and likely reflect the large number of observations. Even more striking than the apparent lack of changes during mucous metaplasia, the number of satellites does not fall in the absence of polymeric mucin synthesis in double deletant mice (Figure 7). Thus, the expression of satellites is apparently specified during secretory cell development and remains constant independent of mucin synthesis. This differs from the expression of satellite number in neurons that depends on neuronal activity (46). The capacity of pre-existing satellites to meet increased mucin synthetic demand is reminiscent of the lack of increase in expression of the exocytic protein Munc18b during mucous metaplasia (47).

De-enrichment of secretory cell satellites for the cis-Golgi marker GM130 (Figures 3, 4, E6) raises the possibility that polymeric mucin synthesis begins in cis-Golgi cisternae in the ribbon, but is then transferred to satellites for further glycosylation and polymerization in medial and trans Golgi cisternae. Supporting this is possible continuity between the ribbon and satellites in the perinuclear region of secretory cells (Figures E5G–I, E8). However, the presence of abundant ER containing incompletely glycosylated mucins in the periphery of secretory cells (Figure E10) supports the alternative possibility that the ribbon is bypassed altogether for mucin synthesis, and that cis-Golgi enzymatic functions reside within satellites despite the absence of the structural protein GM130. In this scenario, the ribbon would instead be the site of synthesis of proteins other than mucins and of lipids. Consistent with biosynthetic roles for peripheral ER and satellites is the presence of numerous adjacent biosynthetic mitochondria (Figures 2, E3, E4). Atypical round mitochondria with few cristae have long been noted in airway secretory cells (45, 48). Recently, it was shown that mitochondria can partition between a classical elongated shape with prominent cristae that is specialized for energy production and a round shape lacking cristae that is specialized for biosynthesis (49, 50). Both types of mitochondria are observed in airway secretory cells, along with mitochondria with an intermediate phenotype (Figures 2A–B, E3, E4). Of interest, synthesis of proline, an abundant amino acid in mucins, is a primary function of biosynthetic mitochondria (49, 50).

There is a clear spatial separation between incompletely glycosylated mucins in the ER and fully glycosylated mucins in secretory granules (Figure E10A–D). The TGN lies immediately adjacent to puncta containing incompletely glycosylated mucins that likely occupy Golgi satellite cisternae (Figure E10E–F). This suggests that the TGN itself is not a site of final mucin synthesis, but rather a site of packaging of fully glycosylated mucins into granules, consistent both with classical views of TGN packaging function (27, 28) and the recent identification of a sorting function for TGN46 (51, 52). Possible signals on mucins of the completion of biosynthesis and readiness for packaging might be the addition of terminal sugars such as sialic acid or fucose, or modifications such as sulfation. Whether enlargement of mucin granules in the progression from immature to mature granules (Figures 2A–B, E4) occurs exclusively from ongoing transfer of mucins from satellites to maturing granules or is also due to lateral fusion between immature granules is not known.

Our study has several limitations, including the following. First, all of our quantitative analyses of satellite expression, localization, and dimensions in mice were performed in the axial bronchus. While this is an intermediate sized airway and therefore likely to be representative of the entire airway tree, it is possible that there are subtle differences in more proximal or more distal airways that were not apparent in our qualitative examinations. We did observe minor differences in satellite distribution between proximal and distal human airway secretory cells (Figures 4, 6). Second, while it seems to be a safe assumption that the satellites we observed by EM correspond to the TGN puncta we observed by IF microscopy, proof would be best obtained by correlative light-electron microscopy (CLEM). However, this technology was not readily available to us. Third, we were unable to prove that the unusual Golgi structure of airway secretory cells is driven by the demands of polymeric mucin synthesis since neither an increase nor decrease in synthesis induced substantial changes in satellite number or dimensions. Support for the hypothesis that mucin synthesis drove the developmental specification of this unusual structure during mammalian evolution might be obtained in future work by showing a correlation between disruption of this structure, through manipulation of the expression of Golgi structural proteins such as golgins or the activity of signaling molecules that specify Golgi structure, and impairment of polymeric mucin synthesis.

In summary, we report an unusual structure of the Golgi apparatus in airway secretory cells. The Golgi apparatus is increasingly recognized as being a site of sensing and scaffolding for numerous cellular processes, including cytoskeletal organization, proteostasis, metabolism, autophagy, inflammation, and apoptosis (29, 33). In view of the postulated roles of these processes in lung pathobiology, further examination is warranted of Golgi apparatus structure and function in airway epithelial secretory cells that are constantly responding to external stimuli and internal immune signals.

## Supplementary Material

Supplementary Files Figure E1. Numerous dispersed TGN elements in mouse airway secretory cells by confocal immunofluorescence microscopy.
Figure E2. Additional images of dispersed TGN in mouse airway secretory cells by deconvolution immunofluorescence microscopy.
Figure E3. Additional EM images of Golgi satellites and ribbons in naïve mouse airway secretory cells.
Figure E4. Additional EM images of Golgi satellites and ribbons in metaplastic mouse airway secretory cells.
Figure E5. Serial block face – scanning electron microscopy (SBF-SEM) of metaplastic mouse airway epithelial cells.
Figure E6. Additional images of the dissociation between cis and trans Golgi cisternae in mouse airway secretory cells.
Figure E7. Localization of cis-Golgi cisternae and TGN with mucin granules in mouse airway secretory cells.
Figure E8. Additional immunofluorescence images of Golgi elements and mucins in human airway secretory cells.
Figure E9. Additional EM images of Golgi satellites in human submucosal gland mucous cells.
Figure E10. Distribution of incompletely and fully glycosylated mucins in human airway secretory cells.
Figure E11. Generation of *Muc5ac/Muc5b* double deletant mice.
Video 1. SBF-SEM and reconstruction of the Golgi apparatus of mouse secretory cells.
Video 2. SBF-SEM of multiple mouse secretory cells demonstrating mucin granules.

This article has a data supplement, which is accessible at the Supplements tab.

## Impact this research will have on clinical medicine and basic science:

Disorders of mucin production and secretion are central to the pathogenesis of multiple muco-obstructive and interstitial lung diseases, as well as lung cancer. However, many details of the post-translational synthesis, intracellular trafficking and packaging of polymeric mucins are unknown. Here we report the discovery of an unusual Golgi apparatus in airway secretory cells that might help in understanding diseases of mucus dysfunction and other epithelial disorders, and in identifying opportunities for therapeutic intervention.
