# Supplementary material for: Airway secretory cells contain both a perinuclear Golgi ribbon and dispersed Golgi satellites
Source: Am J Respir Cell Mol Biol. 2026 Feb 21;74(7):881–95. doi: 10.1093/ajrcmb/aanag018 (PMC13296370; doi:10.1093/ajrcmb/aanag018)
Supplement: aanag018_Supplementary_Data [file aanag018_supplementary_data.zip › aanag018_Supplementary_Data/HoangO-Golgi-Supp-2025-10-20-clean.pdf]

ONLINE DATA SUPPLEMENT

Airway Secretory Cells Contain Both a Perinuclear Golgi Ribbon and Dispersed Golgi Satellites

Oanh N. Hoang, Colin E. Chan, Joshua M. Brenner, Denisse Leza-Rincon, Ana M. Jaramillo, Brendan Dolan, Adam W. Aziz, Rodolfo A. Cardenas, Gerardo J. Cardenas, Eduardo D. Galvez, Joshua B. Hales, Emilia S. Nunez-Pena, Boxuan Yang, Reid T. Powell, Leoncio Vergara, Harry Karmouty-Quintana, Jesper M. Magnusson, Gunnar C. Hansson, Roberto Adachi, John D. Dickinson, Christopher M. Evans, Justin A. Courson, Alan R. Burns, Michael J. Tuvim, Burton F. Dickey

Supplemental Materials and Methods

Materials

All chemicals and supplies were purchased from MilliporeSigma unless indicated otherwise.

Mice

C57BL/6J mice of both sexes were purchased from the Jackson Laboratory and bred to form cohorts. We have found no difference in mucous metaplasia or secretion efficiency between female and male mice from ages 6 to 26 weeks (1), so mice of both sexes within this age interval were used. Mice were housed up to 5 per cage in individually ventilated cages in a specific pathogen-free facility where the exposure to dust was minimized by quarter-inch corn cob bedding, in controlled temperature (21°C) and relative humidity

(55%), and a 12-hour light/dark cycle. Standard chow and water were available ad libitum. The animal care and experimental protocols were approved by the Institutional Animal Care and Use Committee of MD Anderson Cancer Center (MDACC) (permit number 00001214-RN02).

For induction of mucous metaplasia, a single dose of 0.2-4.0 µg mouse recombinant IL-13 (BioLegend) in 40 µl PBS was instilled into the posterior pharynx by direct visualization under isoflurane anesthesia, which mice then aspirated into their lungs. Mice were then sacrificed after 3-5 days. For IF experiments, the dose of IL-13 was 1 µg, for transmission EM experiments the dose was 0.2 µg, and for SBF-SEM the dose was 4 µg. For mice with or without mucous metaplasia, lungs were harvested under tribromoethanol anesthesia, inflated at 20 cm water pressure with 4% neutral buffered paraformaldehyde at 4ºC overnight, dehydrated with graded ethanol, cleared with Histo-Clear (HS-200, National Diagnostics), then embedded in paraffin. A single transverse 5-µm section of the axial bronchus of the left lung was taken from each mouse between the first and second lateral branches as described (1).

For Figure 7, we used tissue from *Muc5ac/Muc5b* double deletant mice that were generated using CRISPR/NHEJ at the University of Colorado (IACUC permit number 46). Gene disruption was achieved by deleting exon 1 as was done previously in embryonic stem cells for both *Muc5ac* (2) and *Muc5b* (3) individually (Fig. E11A). Here, *Muc5ac*/*Muc5b* double knockout mice were produced by targeting intact *Muc5ac* alleles in *Muc5b*$^{lox/lox}$ mice. Guide RNAs 5'-GCTAGTCAATGGCAGTAGTC and 5'-GGCTGGGCATCCAATGTGTG were mixed with Cas9, sperm from *Muc5b*$^{lox/lox}$ males, and

oocytes from C57BL/6 females. The mixture was used to perform in vitro fertilization by Dr. Jennifer Matsuda at National Jewish Health.

F0 pups were screened for *Muc5ac* disruption by PCR using primers 5’-AGCTCAGGGAGAGTCTCAAA (forward) and 5’-AGGCTAAGAGAAACCACATTCC (reverse). Wild type mice yielded an 841 bp amplicon, and mice that had undergone CRISPR with non-homologous end-joining yielded a 332 bp fragment. Sequencing confirmed this to have successfully excised exon 1 from the *Muc5ac* gene. Subsequent breeding confirmed disruption of *Muc5ac* in cis with *Muc5b*^{lox} alleles. *Muc5ac*$^{+/-}$;*Muc5b*$^{lox/+}$ mice were then crossed with CMV-Cre transgenic mice (B6.C-Tg(CMV-cre)1Cgn/J, Strain #006054, Jackson Laboratory) to produce *Muc5b* knockout alleles in all tissues, including the germline. Heterozygotes also carrying Cre transgenes were crossed with C57BL/6J mice (Jackson Laboratory), and resulting *Muc5ac*$^{+/-}$; *Muc5b*$^{+/-}$ mice were bred again with C57BL/6J mice to confirm germline inheritance independent of continued CMV-Cre transgene presence. Confirmation of *Muc5ac* gene disruption was validated using RT-qPCR on stomach tissues, which demonstrated high levels of baseline *Muc5ac* transcripts in *Muc5ac*$^{+/+}$;*Muc5b*$^{lox/lox}$ mice and *Muc5ac* mRNA absence in *Muc5ac*$^{-/-}$; *Muc5b*$^{-/-}$ littermates (Fig. E11B).

Muc5ac$^{+/-}$;*Muc5b*$^{+/-}$ mice were bred together to produce experimental animals used here. Notably, there was a strong effect of double deficiency that was apparent at all stages of life. Due to their linkage on mouse chromosome 7, intercrossing *Muc5ac*$^{+/-}$;*Muc5b*$^{+/-}$ mice was expected to yield simple Mendelian inheritance ratios. Double knockout pups were observed born at only 8.1% frequencies ($p < 0.0001$, chi square). By contrast, *Muc5ac* and *Muc5b* single knockout lines produced homozygous deletants at 23% and 21%

frequencies (p = 0.136 and 0.054, respectively). In addition to these observations in early life, there were also significant effects of combined *Muc5ac* and *Muc5b* gene deficiency on post-natal survival. Tracking cohorts of mice over one year revealed significant early mortality in single *Muc5b* knockout mice, consistent with what was reported previously (3), and deficiency in both mucins caused even greater survival impairment (Fig. E11C). Median survival in *Muc5ac*$^{-/-}$; *Muc5b*$^{-/-}$ mice was only 6.6 weeks, which was significantly lower than 32.6 weeks median survival in *Muc5b*$^{-/-}$ mice (p = 0.0004).

Human tissue

For examination of most human airways, de-identified tissue from lungs donated but not used for transplantation was obtained by the Pulmonary Center of Excellence Biobank at the University of Texas Health Science Center at Houston under an approved institutional review board protocol (HSC-MS-08-0354). None of the donors had known lung disease (Table S1). For proximal airways, bronchial tissue was dissected free from major vessels and pleural tissue. A 1-1.5 cm longitudinal piece of lobar or segmental bronchus near the midpoint between its take-off and its termination was excised and placed in 10% neutral buffered formalin for 24 h at 4ºC overnight, then embedded in paraffin. For distal airways, a section was made in the peripheral lung parallel to the pleural surface, 1-2 cm from the pleura, then fixed and embedded as above. Airways 200-1,000 µm in diameter and lacking

Table S1. Sources of human airway tissue at the University of Texas.

Study #	Age	Sex	Height	Weight	Lung	Lung dx	Smoking	Cause of death
1065	35	M	178	95	R	None	Unknown	Brain anoxia 2° drug intoxication
1066	21	F	177	67	R	None	Yes	Head trauma
1069	20	M	180	112	R	None	Unknown	Head trauma
1071	29	M	170	70	L	None	No	Brain anoxia 2° drug intoxication

Height is in cm; **Weight** is in kg. Abbreviations: **Lung dx** = known lung disease; **R** = right; **L** = left.

submucosal glands were then selected for microscopic analysis at MDACC.

For the imaging of immature mucins in Figure E10, normal human tracheal tissue was obtained from transplant donor tissue collected at the Sahlgrenska University Hospital, Gothenburg, Sweden following protocols approved by the Swedish Ethical Review Authority (Etikprövningsmyndigheten, 2020-02658). The tissue was fixed overnight in 4% neutral buffered formalin and then embedded in paraffin.

Immunofluorescence microscopy of mouse and human airways

For imaging of mouse and human airways at MDACC, paraformaldehyde-fixed paraffin-embedded tissue blocks were cut into sections 5 μm thick, deparaffinized with xylene, and rehydrated with ethanol/water. Samples were then placed in a 10 mM sodium citrate bath (pH 6.0) and heated in a pressure cooker for 10 min. After cooling, lung sections were washed with PBS and permeabilized with 0.3% Triton X-100 in PBS for 20 min. Sections were then blocked with 5% normal donkey serum (NDS) in PBS and 0.05% Tween 20 (PBST) for 1 h at room temperature. Following PBS and PBST washes, background autofluorescence was quenched with an electrostatic agent (Vector TrueVIEW) for 3 min. Sections were given a final PBS wash and mounted with Fluorescence Mounting Medium (Dako). Antibodies used for immunofluorescence microscopy are listed in Table S2.

Table S2. Antibodies used for immunofluorescence microscopy.

**Antibodies**	**Source**	**Identifier**
Primary antibodies		
Mouse monoclonal anti-MUC5AC	ThermoFisher	45M1, # MA5-12178
Mouse monoclonal anti-MUC5AC DyL550 conjugate	Novus Biologicals	45M1, # NBP2-32732R

Rabbit polyclonal anti-MUC5B	Ehre laboratory	UNC222 (4)
Rabbit polyclonal anti-human MUC5B	MilliporeSigma	# HPA008246
Rabbit polyclonal anti-human MUC5B	Hansson laboratory	MUC5B-D3 (5)
Mouse monoclonal anti-human iMUC5AC*	Clausen laboratory	CLH2 (6)
Mouse monoclonal anti-human iMUC5B[†]	Clausen laboratory	PANH2 (7)
Mouse monoclonal anti-AcTubulin	Sigma-Aldrich	T6793
Goat polyclonal anti-uteroglobin	EMD Millipore	ABS1673
Rat monoclonal anti-human uteroglobin	R&D Systems	MAB4218
Goat polyclonal anti-mouse peptidase inhibitor 16	R&D Systems	AF4929
Rabbit polyclonal anti-MEOX2	Novus Biologicals	NBP2-30647
Rabbit polyclonal anti-mouse TGN46	Abcam	ab16059
Sheep polyclonal anti-human TGN46	Bio-Rad	AHP500GT
Rabbit monoclonal anti-TGN46	ThermoFisher	JF1-024, # MA5-32532
Rabbit polyclonal anti-GRASP55	Novus Biologicals	NBP1-89747
Mouse monoclonal anti-GM130	BD Biosciences	610822
Rabbit polyclonal anti-calnexin	Abcam	ab22595
Secondary antibodies		
Donkey anti-mouse, Alexa 488	ThermoFisher	# A-21202
Donkey anti-mouse, Alexa 555	ThermoFisher	# A-31570
Donkey anti–mouse, Alexa 647	ThermoFisher	# A-31571
Donkey anti-rabbit, Alexa 488	Jackson Immuno	# 715-545-152
Donkey anti-rabbit, Cy3	Jackson Immuno	# 711-165-152
Donkey anti-rabbit, Alexa 647	ThermoFisher	# A-31573
Donkey anti-rabbit, Alexa 555	ThermoFisher	# A-31572
Donkey anti-goat, Alexa 647	Jackson Immuno	# 705-605-147
Rabbit anti-sheep, unconjugated	Jackson Immuno	# 313-001-003
If antibody is known to be species-specific towards its target, this is indicated. *iMUC5AC = incompletely glycosylated MUC5AC, [†]iMUC5B= incompletely glycosylated MUC5B.		

For enumeration of TGN elements, a pilot study was performed using a confocal microscope (Nikon A1, Japan) with a 60x lens (Apochromat TIRF 60XO, NA 1.49, Nikon) with oil immersion (Figure E1). Settings were optimized using the Nyquist rate. Z-stacked optical sections were evaluated for TGN46 puncta in 0.5 μm steps to avoid counting the same structure twice, and cell borders were determined from differential interference contrast images. For all subsequent studies except Figure E10, widefield fluorescence

microscopy with post-acquisition deconvolution of the images was performed as follows. Z-stacked images of 0.5 µm steps were taken on a DeltaVision Elite microscope (GE Healthcare) using a 100x oil immersion objective (UPLSAPO 100XO 1-U2B836, NA 1.4, Olympus) and 0.23 µm optical sections. Images were then deconvolved with SoftWoRx software (GE Healthcare). For enumeration of TGN elements (Figure 1C), TGN46 puncta were counted on optical sections 1 µm apart.

For imaging of immature mucins in human tissue at the University of Gothenburg, formalin-fixed, paraffin-embedded tissue blocks were cut into sections 6 µm thick, deparaffinized with xylene, rehydrated in a series of graded ethanol baths, followed by antigen retrieval in 10 mM citrate buffer (pH 6.0). Following this, sections were permeabilized with 0.1% Triton X-100 in TBS for 10 min and then blocked with 1% bovine serum albumin/10% donkey serum in TBS for 1 h at room temperature. Afterwards, sections were incubated sequentially with primary antibodies raised against mature MUC5AC (clone 45M1), mature hMUC5B (MUC5B-D3 antibodies), incompletely glycosylated MUC5AC (iMUC5AC, clone CLH2), incompletely glycosylated MUC5B (iMUC5B, clone PANH2), TGN46, and calnexin as detailed in Table S2. Antibodies against iMUC5AC were raised against a non-glycosylated hMUC5AC peptide, and antibodies against iMUC5B were raised against purified hMUC5B deglycosylated with trifluoromethanesulfonic acid. Following incubation with primary antibodies, sections were washed and incubated with appropriate fluorophore-conjugated secondary antibodies as detailed in Table S2. Cell nuclei were counterstained with Hoechst 34580 (5 µg/ml in TBS). High resolution confocal micrographs were acquired on an upright LSM900 confocal microscope equipped with an Airyscan2 detector (Carl Zeiss) using a Pan-Apochromat 63x/1.4 oil DIC M27 lens. Confocal

micrographs were captured using Zen software (Zen blue v3.1; Carl Zeiss), data was exported to Imaris (v9.5; Bitplane), and images were exported in TIFF format.

Transmission electron microscopy

For studies in mice, mild mucous metaplasia was induced with a single dose of 0.2 µg IL-13 instilled intrapharyngeally as described above, then mice were anesthetized and sacrificed five days later. Lungs were excised and fixed in 2.5% glutaraldehyde in 0.1 M sodium cacodylate buffer (pH 7.2) containing 20 mM calcium chloride for 2 h, followed by a 1-h secondary fixation in buffered 1% osmium tetroxide. The fixed left lung was then sectioned into a single transverse cut of the axial airway between lateral branches 1 and 2 and embedded in EMbed 812 epoxy resin (14120, Electron Microscopy Sciences). Sections 100 nm thick were stained with uranyl acetate and lead citrate and were viewed in a Tecnai 12 transmission electron microscope. Secretory airway epithelial cells were identified by an apical plasma membrane contacting the airway lumen, the presence of secretory granules, and absence of cilia. Cells were selected for further imaging if a complete plasma membrane was visible that contacted the airway lumen, neighboring cells on both sides, and the interstitium on the basal surface, and if a portion of the nucleus at least 2 µm wide was visible. Multiple photographs of each secretory cell were assembled using Adobe Photoshop into photomontages containing the complete cell. These composite images, termed “cellular profiles”, were then analyzed for the distribution of Golgi elements, which were identified as stacks of membrane-bound cisternae (8). To measure the position of a satellite on the apical-basal axis, a line from the middle of the nucleus to the middle of the apical membrane (NA) was set as +1, and the distance from the middle of the nucleus to the satellite (NS) was calculated as a fraction (Figure 2C).

Satellites below the middle of the nucleus were calculated as negative fractions. To measure the position of a satellite on the lateral axis, a line from the central apical-basal axis of the cell to the lateral membrane (CL) was set as +1, and the distance from the central apical-basal axis to the satellite (CS) was calculated as a fraction (Figure 2D).

For routine studies of human tissue, fixation and staining were as described above for mouse tissue. To highlight Golgi elements in some studies, glutaraldehyde-fixed tissue was stained with Zinc Iodide-Osmium tetroxide reagent (ZIO) according to (9), with minor deviations. Briefly, 3 g of Zn powder (Sigma #243469) were suspended in 20 ml of water, then 3 g of I_2 flakes (Sigma # 376558) were added gradually with vigorous mixing to prevent overheating. The suspension was then filtered, and 4 ml of the colorless solution were mixed with 2 ml 50 mM Tris (pH 7.4) and 2 ml 2% OsO_4 in water, resulting in a light brown solution. Tissue samples were immersed in this solution for more than 100 h in the dark at 4°C. For studies in cultured human airway epithelial cells at both the University of Nebraska (Figure 5C) and MDACC (Figure 5D-F), non-diseased human airway epithelial cells (HAECs) were derived for culture from excess airway tissue donated for lung transplantation as previously described (10). After 5-7 days of expansion with BEGM media (Lonza #CC-3170), air-liquid interface (ALI) conditions were established and HAECs were fed thereafter with PneumaCult ALI media. Beginning 14 days after the establishment of ALI, cells were treated 7 days with IL-13 (10 ng/mL), then fixed. Cells cultured at the University of Nebraska were fixed with 2% paraformaldehyde and 2.5% glutaraldehyde in 100 mM cacodylate buffer (pH 7.2) for 2 h at room temperature, followed by a 1-h secondary fixation in buffered 1% osmium tetroxide. They were viewed on a JEOL 1200 EX electron microscope equipped with an AMT 8 megapixel digital camera

(Advanced Microscopy Techniques). Cells cultured at MDACC (Figure 5D-F) were stained with ZIO and viewed on a Tecnai 12 electron microscope as above.

Serial block face – scanning electron microscopy (SBF-SEM)

This was performed on mouse tissue with mucous metaplasia induced with a single dose of 4 μg IL-13 instilled intrapharyngeally as described above. Tissue was fixed with glutaraldehyde, impregnated with heavy metals, and prepared for SBF-SEM as previously described (11). Serial imaging was performed using a 3View®2 system (Gatan) mounted in a MIRA3 field emission scanning electron microscopy (TESCAN) under high vacuum with a Schottky emitter and an accelerating voltage of 9 kV with spot size of 4.3 nm and pixel size ranging from 2.7-5.9 nm. Backscatter electron detection was used to image the block face. Serial images were acquired at 100 nm intervals. Image stacks were post-processed for spatial drift removal using Gatan DigitalMicrograph software. Segmentation and reconstruction of the Golgi apparatus from a secretory cell was performed using Amira 6.0.1 software (FEI Systems) as previously described (12). In brief, the nucleus was reconstructed along its surrounding nuclear envelope, the Golgi apparatus by both its electron dense staining and cisternal processes, and the associated secretory granule along its limiting membrane based on the electron density of its lumenal content as described in the legend to Figure E5G.

Data Analysis

Statistical analyses were performed and plots generated using GraphPad Prism version 8.4.3 (Prism software), with $P < 0.05$ considered significant. Methods of analysis, P values and n values for each sample are included in figure legends.

Supplemental Results

Calculation of the number of Golgi satellites per mouse airway secretory cell visible by electron microscopy (related to Figure 2E). From a sample of EM cellular profiles of both naïve and metaplastic airway secretory cells (five each), we found an average cell width of 10.4 μm and height of 16 μm. Assuming that a cell has a cylindrical shape, the average cross-sectional area (3.14 x radius 5.2 μm^2) is 84.9 μm^2, and volume (84.9 μm^2 x height 16 μm) is 1,358 μm^3. Further assuming that an EM image is roughly vertical on an apical-basal axis, passes through the center of a cross-sectional circle, and is seen en face, the average cross-sectional area (width 10.4 μm x height 16 μm) is 166.4 μm^2, and volume (area 166.4 μm^2 x section thickness 0.1 μm) is 16.6 μm^3. Based on these assumptions, an EM image would sample 16.6 μm^3/1,358 μm^3 (0.012%) of the cell volume. With a mean of 0.68 satellites visualized in naïve cells per EM image, the mean total satellite number per cell is estimated at 55.5, and with a mean of 0.71 satellites visualized in metaplastic cells, the mean total satellite number per cell is estimated at 57.9.

Supplemental References

1. Jaramillo AM, Piccotti L, Velasco WV, Delgado ASH, Azzegagh Z, Chung F, Nazeer U, Farooq J, Brenner J, Parker-Thornburg J, Scott BL, Evans CM, Adachi R, Burns AR, Kreda SM, Tuvim MJ, Dickey BF. Different Munc18 proteins mediate baseline and stimulated airway mucin secretion. *JCI Insight* 2019; 4.
2. Hasnain SZ, Evans CM, Roy M, Gallagher AL, Kindrachuk KN, Barron L, Dickey BF, Wilson MS, Wynn TA, Grencis RK, Thornton DJ. Muc5ac: a critical component mediating the rejection of enteric nematodes. *J Exp Med* 2011.
3. Roy MG, Livraghi-Butrico A, Fletcher AA, McElwee MM, Evans SE, Boerner RM, Alexander SN, Bellinghausen LK, Song AS, Petrova YM, Tuvim MJ, Adachi R, Romo I, Bordt AS, Bowden MG, Sisson JH, Woodruff PG, Thornton DJ, Rousseau K, De la Garza MM, Moghaddam SJ, Karmouty-Quintana H, Blackburn MR, Drouin

SM, Davis CW, Terrell KA, Grubb BR, O'Neal WK, Flores SC, Cota-Gomez A, Lozupone CA, Donnelly JM, Watson AM, Hennessy CE, Keith RC, Yang IV, Barthel L, Henson PM, Janssen WJ, Schwartz DA, Boucher RC, Dickey BF, Evans CM. Muc5b is required for airway defence. *Nature* 2014; 505: 412-416.
4. Ehre C, Worthington EN, Liesman RM, Grubb BR, Barbier D, O'Neal WK, Sallenave JM, Pickles RJ, Boucher RC. Overexpressing mouse model demonstrates the protective role of Muc5ac in the lungs. *Proc Natl Acad Sci U S A* 2012; 109: 16528-16533.
5. Fakih D, Rodriguez-Pineiro AM, Trillo-Muyo S, Evans CM, Ermund A, Hansson GC. Normal murine respiratory tract has its mucus concentrated in clouds based on the Muc5b mucin. *Am J Physiol Lung Cell Mol Physiol* 2020; 318: L1270-L1279.
6. Reis CA, David L, Nielsen PA, Clausen H, Mirgorodskaya K, Roepstorff P, Sobrinho-Simoes M. Immunohistochemical study of MUC5AC expression in human gastric carcinomas using a novel monoclonal antibody. *Int J Cancer* 1997; 74: 112-121.
7. Nielsen PA, Mandel U, Therkildsen MH, Clausen H. Differential expression of human high-molecular-weight salivary mucin (MG1) and low-molecular-weight salivary mucin (MG2). *J Dent Res* 1996; 75: 1820-1826.
8. Wei JH, Seemann J. Golgi ribbon disassembly during mitosis, differentiation and disease progression. *Curr Opin Cell Biol* 2017; 47: 43-51.
9. Johkura K, Usuda N, Tanaka Y, Fukasawa M, Murata K, Noda T, Ohno N. Whole-cell observation of ZIO-stained Golgi apparatus in rat hepatocytes with serial block-face scanning electron microscope, SBF-SEM. *Microscopy (Oxf)* 2022; 71: 262-270.
10. Sweeter JM, Kudrna K, Hunt K, Thomes P, Dickey BF, Brody SL, Dickinson JD. Autophagy of mucin granules contributes to resolution of airway mucous metaplasia. *Sci Rep* 2021; 11: 13037.
11. Courson JA, Landry PT, Do T, Spehlmann E, Lafontant PJ, Patel N, Rumbaut RE, Burns AR. Serial Block-Face Scanning Electron Microscopy (SBF-SEM) of Biological Tissue Samples. *J Vis Exp* 2021.
12. Courson JA, Rumbaut RE, Burns AR. Impact of Obesity and Age on Mouse Corneal Innervation at the Epithelial-Stromal Interface. *Invest Ophthalmol Vis Sci* 2024; 65: 11.

Supplemental Figure Legends

Figure E1. Numerous dispersed TGN elements in mouse airway secretory cells by confocal immunofluorescence microscopy. Laser confocal micrographs of mouse airways stained with antibodies against MUC5AC, MUC5B, and TGN46, with nuclei stained with DAPI, and merged with differential interference contrast (DIC) images. (*A*) Naïve, uninflamed airway with no visible MUC5AC staining. Secretory cells are marked by MUC5B staining. Dashed box outline in top left image shows region of interest magnified in dual channel fluorescence views without DIC in left three panels of bottom row. The total number of TGN46 in two ciliated and two secretory cells, measured as described in Methods, is shown in the top right panel. (*B*) Airway with mucous metaplasia due to instillation of 1 µg IL-13 showing MUC5AC staining. Layout of the panels is as in *A*.

Figure E2. Additional images of dispersed TGN in mouse airway secretory cells by deconvolution immunofluorescence microscopy. Immunostaining is exactly as in Figure 1 using antibodies against CCSP to mark secretory cells, acetylated tubulin (AcTub)

to mark ciliated cells, TGN46 to mark the trans-Golgi network, and DAPI to stain nuclei in the axial bronchi of mice. (*A*) Three naïve airways without mucous metaplasia. (*B*) Three airways with mucous metaplasia due to instillation 1 µg IL-13.

Figure E3. Additional EM images of Golgi satellites and ribbons in naïve mouse airway secretory cells. Cell profiles compiled from multiple electron micrographs of secretory cells as in Figure 2. (*A*) The same cellular profile shown in Figure 2A is enlarged and annotated here. The Golgi ribbon is outlined in magenta and a Golgi satellite is outlined in orange. SG = secretory granule, MVB = multivesicular body, eM = mitochondrion specialized for energy production, sM = mitochondrion specialized for synthesis, iM = mitochondrion intermediate between energy and synthesis specialization, L = lysosome, N = nucleus. (*B*) Another secretory cell profile annotated as in *A*. (*C-E*) Additional secretory cell profiles not annotated.

Figure E4. Additional EM images of Golgi satellites and ribbons in metaplastic mouse airway secretory cells. Cell profiles compiled from multiple electron micrographs of secretory cells as in Figure 2. (*A*) The same cellular profile shown in Figure *2B* is enlarged and annotated here. The Golgi ribbon is outlined in magenta and two Golgi satellites are outlined in orange. The dashed teal lines mark boundaries between the apical third of the cell filled with mature mucin granules, the middle third filled with immature mucin granules, and the basal third mostly devoid of mucin granules. Mature mucin granules are larger than immature granules, more electron dense, rounder, and often contain a small spherical region of differing electron density that is uniformly observed in serial block face – scanning electron microscopy (SBF-SEM) (red arrowhead in Figure E5J-L; Video 2). mG = mature mucin granule, iG = immature mucin granule, eM = mitochondrion specialized for energy production, sM = mitochondrion specialized for synthesis, iM = mitochondrion intermediate between energy and synthesis specialization, L = lysosome, N = nucleus. (*B*) Another secretory cell profile partially annotated as in *A*. (*C*) An additional secretory cell profile annotated as in *A*, but with only a single boundary marked between the apical pole containing mature mucin granules and the middle region of the cell filled with immature mucin granules and mitochondria.

Figure E5. Serial block face – scanning electron microscopy (SBF-SEM) of metaplastic mouse airway epithelial cells. Tissue from mice with mucous metaplasia induced by instillation of 4 µg IL-13 into the airway was fixed and processed for SBF-SEM as described in Methods. Videos were constructed from the serial scanning EM images, with panels *D-F* taken from Video 1 and panels *J-L* from Video 2. (*A*) A high resolution image not from Video 1 or 2 showing Golgi stacks, mucin granules, mitochondria, and portions of two nuclei. (*B*) The boxed area in A is shown at higher magnification. Green arrowheads point to Golgi satellites, blue arrows point to immature mucin granules, and red arrow points to a maturing mucin granule. (*C*) The boxed area in *B* is shown at higher magnification. (*D*) An image corresponding to Video 1 at 7 seconds showing a Golgi ribbon (green arrowheads) just above the cell's nucleus (left). (*E*) An image from a section close to that in *D* showing Golgi stacks (green arrowheads) among immature mucin granules. (*F*) An image from a section adjacent to that in *E* showing more Golgi stacks (green arrowheads), with one of these adjacent to an immature mucin granule (red asterisk). (*G*) Reconstruction from multiple sections of a portion of the Golgi apparatus of a mouse secretory cell superimposed on the image shown in *F*. The nucleus is shown in blue, Golgi

elements in green, and the immature secretory granule in red. (*H*) Reconstruction of different sections from those in *G* of the nucleus and Golgi apparatus superimposed on the same image. (*I*) The reconstructed nucleus and Golgi apparatus shown in isolation and rotated. It appears that the Golgi ribbon and nearby satellites may be connected, though the segmentation is not at sufficient resolution to be certain. (*J*) An image corresponding to Video 2 at 11 seconds showing four secretory cells containing mucin granules and one ciliated cell with a prominent ciliary tuft. Mature mucin granules near the cell apices contain electron-dense spherical regions, usually in the center of the granule (red arrowhead). Immature mucin granules are relatively electron-lucent (blue arrow), but maturing granules can show intermediate electron density and electron-dense central regions (red arrow). There is extensive endoplasmic reticulum (blue arrowhead) among the immature mucin granules One of the secretory cells, outlined with a dashed yellow line, appears to be undergoing exocytosis, with swelling of the central spherical region. (*K-L*) Images from sections at 13 and 18 seconds with the same annotation scheme as in *J*.

Figure E6. Additional images of the dissociation between cis and trans Golgi cisternae in mouse airway secretory cells. *(A)* Immunostaining and microscopy are exactly as in Figure 3 using antibodies against CCSP to mark secretory cells, GM130 to mark cis-Golgi cisternae, GRASP55 to mark trans-Golgi cisternae, and DAPI to mark nuclei in the axial bronchus of a mouse with mucous metaplasia due to treatment with 1 µg IL-13. Fluorescence image is superimposed on a differential interference contrast (DIC) image. *(B)* As in *A*, but in a naïve mouse without mucous metaplasia, and without DIC image. *(C)* As in *A*, in another mouse with mucous metaplasia, without DIC image.

Figure E7. Localization of cis-Golgi cisternae and TGN with mucin granules in mouse airway secretory cells. *(A)* Immunofluorescence deconvolution microscopy using antibodies against CCSP to mark secretory cells, GM130 to mark cis-Golgi cisternae, MUC5B to mark mucin granules, and DAPI to mark nuclei in the axial bronchus of a naïve mouse airway. *(B)* Microscopy as in *A*, but in an airway with mucous metaplasia due to treatment with 1 µg IL-13. *(C-D)* Microscopy as in *A-B*, but using TGN46 rather than GM130. *(E-F)* Microscopy as in *C-D* but using antibodies against MUC5AC rather than MUC5B.

Figure E8. Additional immunofluorescence images of Golgi elements and mucins in human airway secretory cells. *(A)* Immunostaining and fluorescence microscopy as in Figure 4, but using antibodies against MUC5AC and MUC5B, together with antibodies against TGN46 to mark the trans-Golgi network and DAPI to mark nuclei in a proximal human airway. The fluorescent image is merged with a differential interference contrast (DIC) image. *(B)* Microscopy as in *A*, but in a distal human airway. *(C)* Two additional images of proximal human airways as in *A*, but without DIC images.

Figure E9. Additional EM images of Golgi ribbons and satellites in human submucosal gland mucous cells. Human airway tissue was fixed and stained with ZIO to highlight Golgi elements, as in Figure 5D. (*A*) A Golgi ribbon (green arrow) is seen extending from the nucleus alongside mucin granules, and Golgi satellites (red arrowhead) are seen among the granules. (*B-C*) Additional Golgi ribbons and satellites are indicated as in *A*.

Figure E10. Distribution of incompletely and fully glycosylated mucins in human airway secretory cells. (*A-B)* Immunofluorescence Airyscan microscopy of normal human tracheal tissue using antibodies against incompletely glycosylated mucin proteins (iMUC5AC and iMUC5B), and against fully glycosylated and folded mucin proteins (fMUC5AC and fMUC5B) to mark granules. Hoechst stain (grey) marks nuclei. *(C-D)* Immunofluorescence microscopy as in *A-B*, but with antibodies against calnexin to mark ER instead of antibodies against fully glycosylated mucins. Yellow arrowhead points to black circle occupied by mucin granule. *(E-F)* Immunofluorescence microscopy as in *A-B*, but with antibodies against TGN46 to mark the trans-Golgi network instead of antibodies against fully glycosylated mucins. Yellow arrowhead points to black circle occupied by mucin granule.

Figure E11. Generation of *Muc5ac/Muc5b* double deletant mice. *(A)* The *Muc5b* gene (right panel) was previously targeted to insert *loxP* sites (red flags) flanking the first exon. The gene structure is shown at low resolution in the top figure, at higher resolution in the middle figure, and after Cre-mediated recombination in the bottom figure. Mice bearing the floxed *Muc5b* allele were used for CRISPR/NHEJ-mediated excision of the first exon of *Muc5ac* (left panel). The location of primers used for genotyping is indicated by single-sided arrows. *(B) Muc5ac* transcripts relative to transcripts of the *Hprt1* housekeeping gene in the stomach of double deletant mice were compared to those in *Muc5b*$^{lox/lox}$ mice in which the *Muc5ac* gene was intact. (*C*) Post-natal survival is plotted of mice with intact or deletant alleles of *Muc5ac* and *Muc5b*.

Video 1. SBF-SEM and reconstruction of the Golgi apparatus of a mouse secretory cell. This video shows to good advantage the apposition of Golgi satellites to immature mucin granules. See Figure E5A-F for annotation.

Video 2. SBF-SEM of multiple mouse secretory cells demonstrating ultrastructure of mucin granules. This video shows the essentially universal presence of a small electron-dense spherical region within mature mucin granules that helps distinguish mucin granules from round biosynthetic mitochondria. See Figure E5J-L for annotation.
