# Supplementary material for: Airway secretory cells contain both a perinuclear Golgi ribbon and dispersed Golgi satellites
Source: Am J Respir Cell Mol Biol. 2026 Feb 21;74(7):881–95. doi: 10.1093/ajrcmb/aanag018 (PMC13296370; doi:10.1093/ajrcmb/aanag018)
Supplement: aanag018_Supplementary_Data [file aanag018_supplementary_data.zip › aanag018_Supplementary_Data/HoangO-Golgi-Supp-Figures-2025'11'06.pdf]

Figure E1

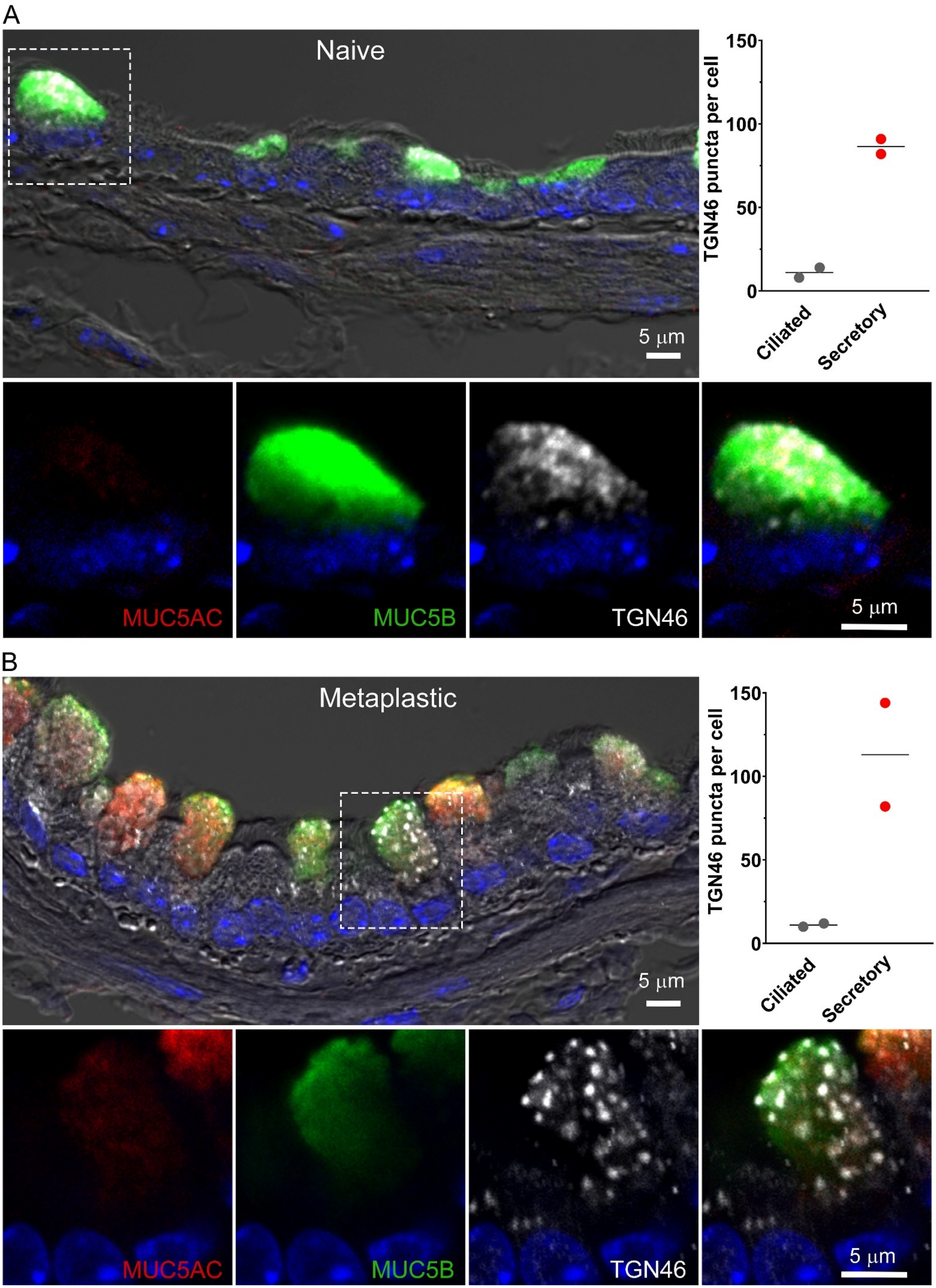
A
Naive
5 μm
TGN46 puncta per cell
150
100
50
0
Ciliated
Secretory
MUC5AC
MUC5B
TGN46
5 μm
B
Metaplastic
5 μm
TGN46 puncta per cell
150
100
50
0
Ciliated
Secretory
MUC5AC
MUC5B
TGN46
5 μm

Figure E2

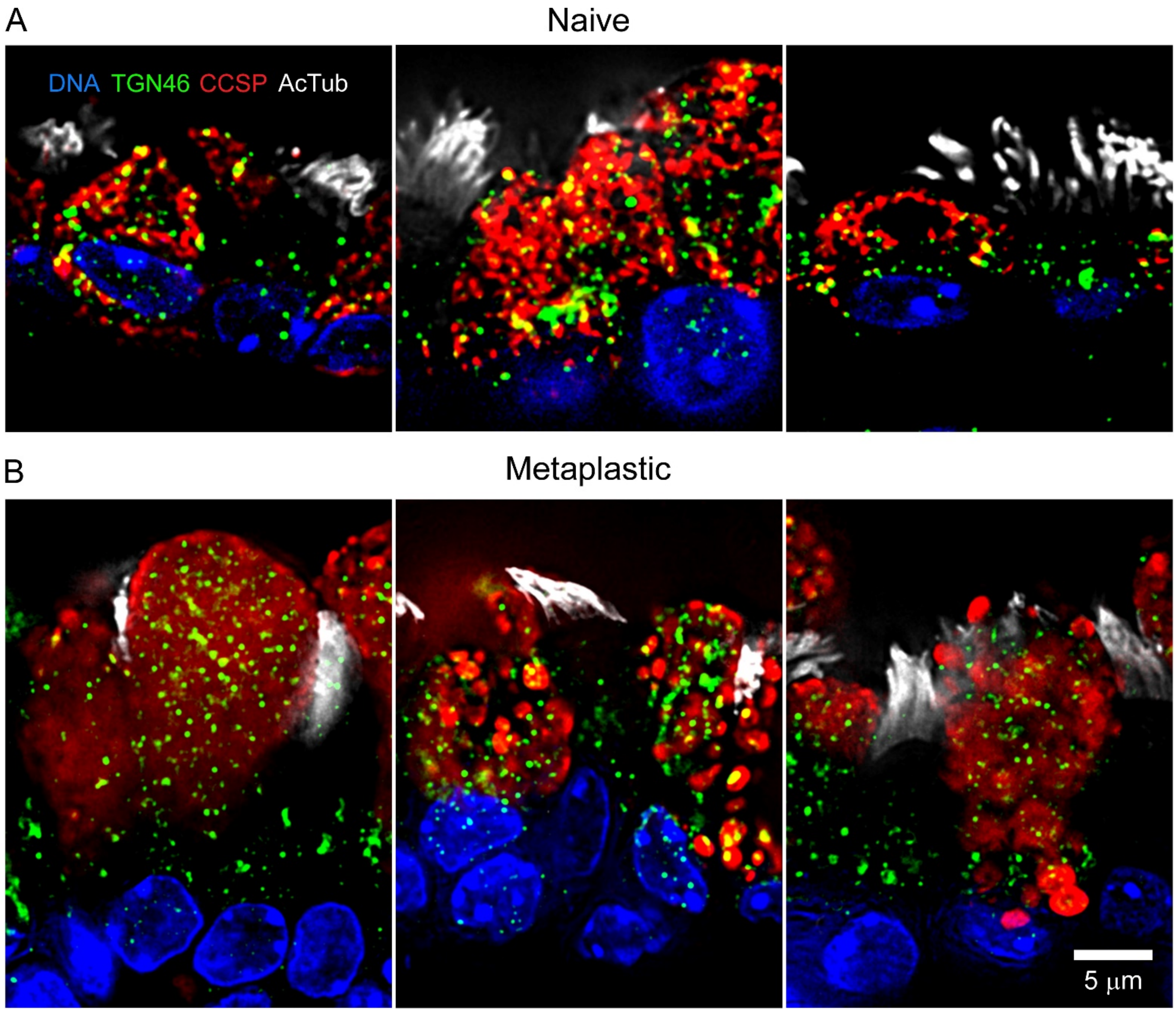

Figure E3

A

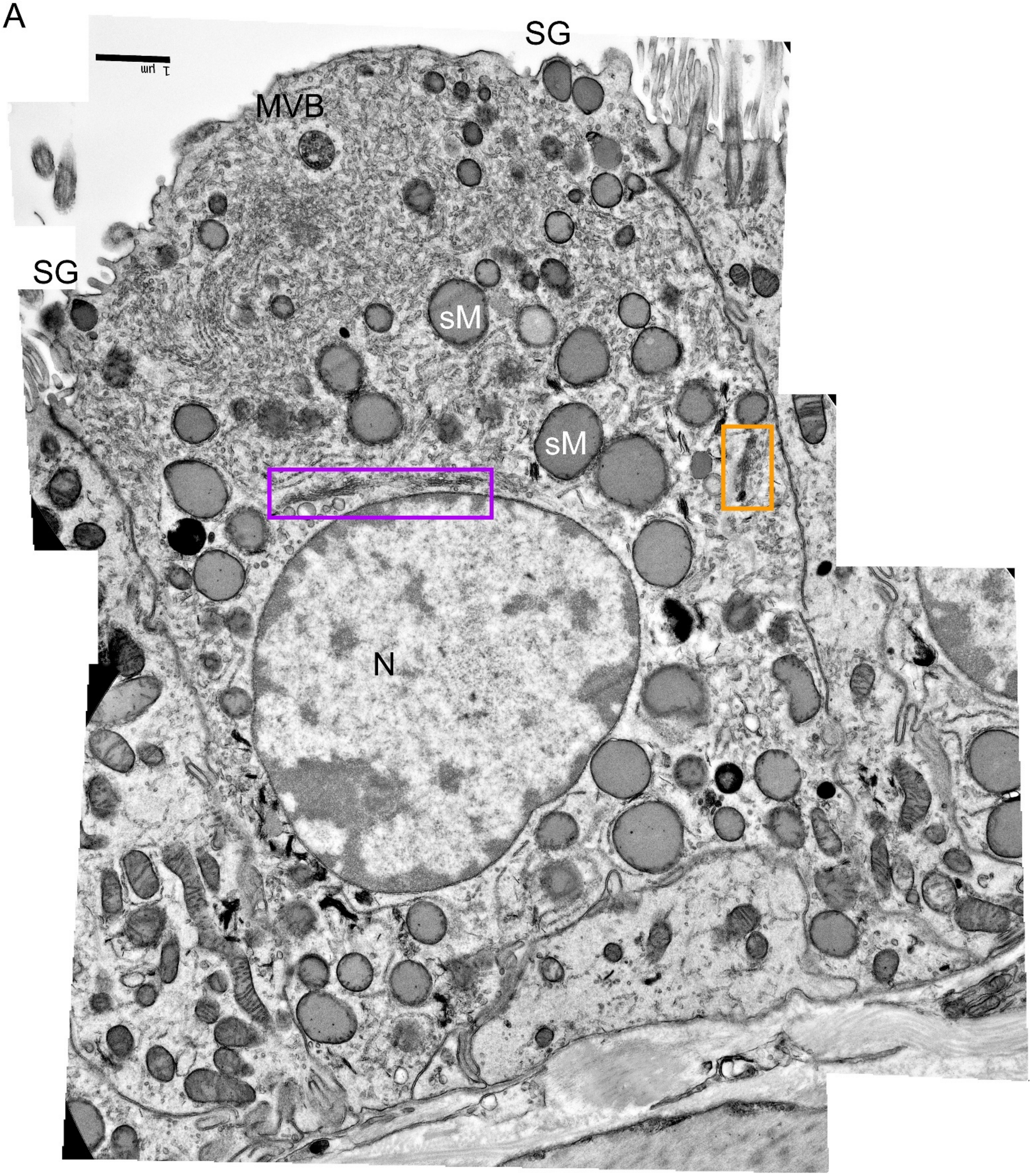

B

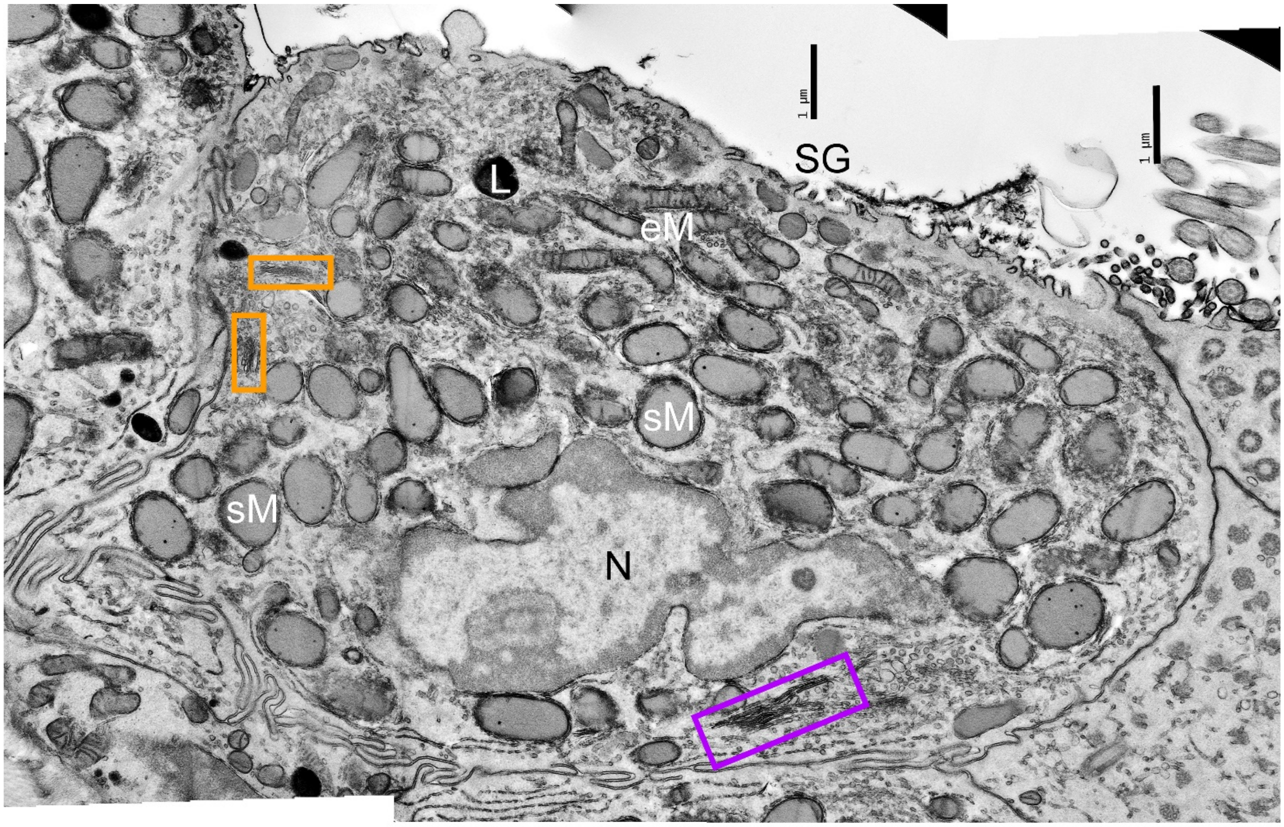

C

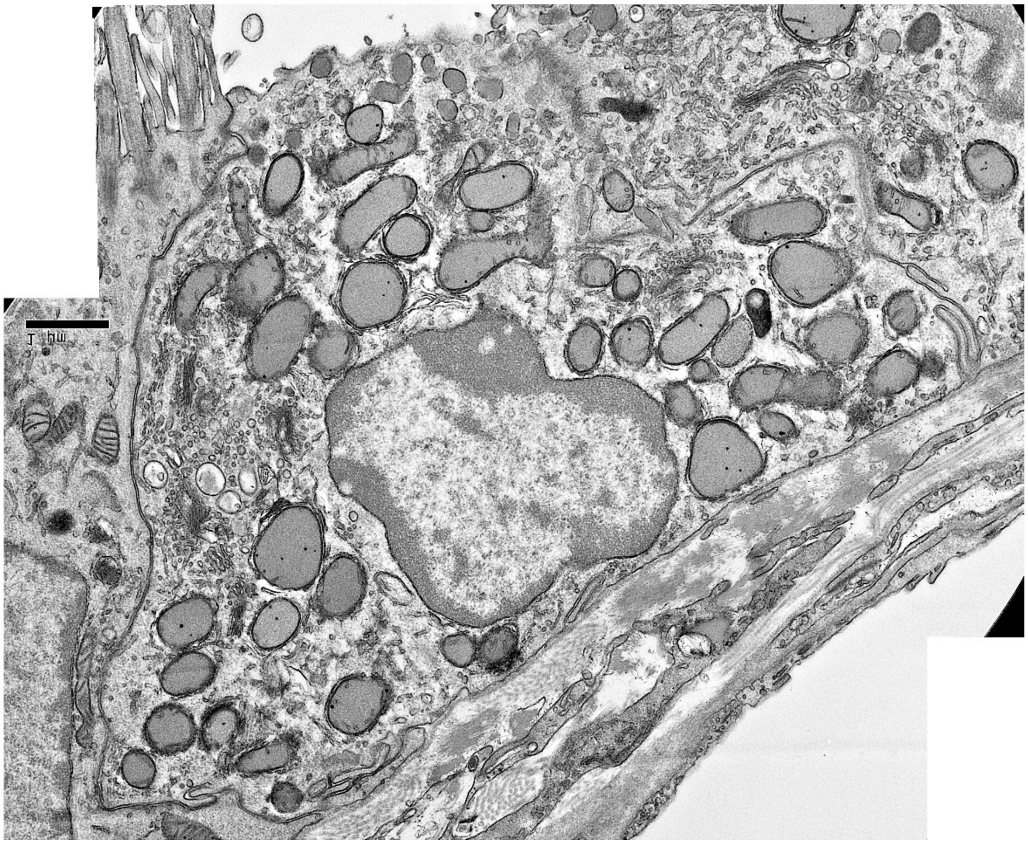

D

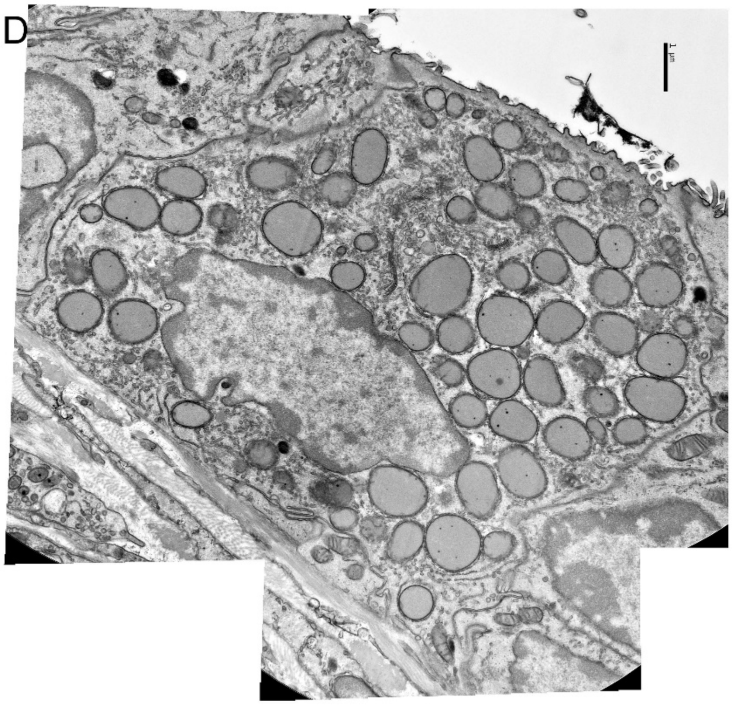

E

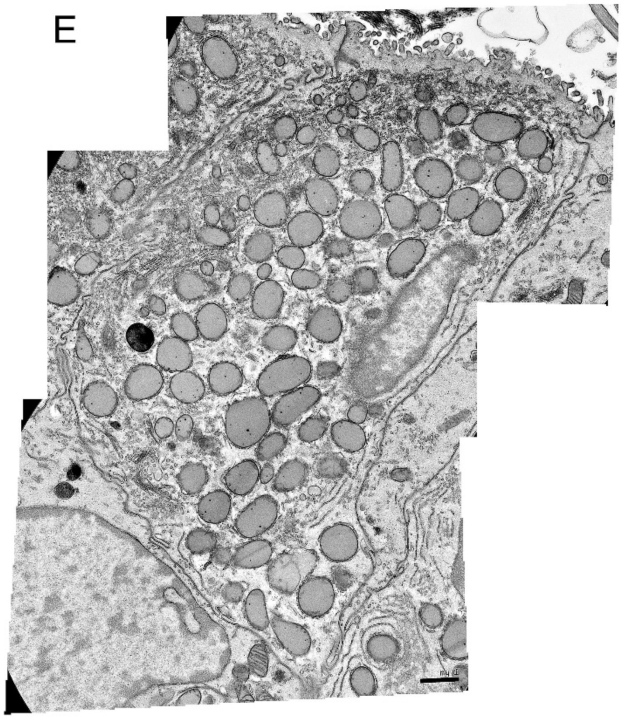

F

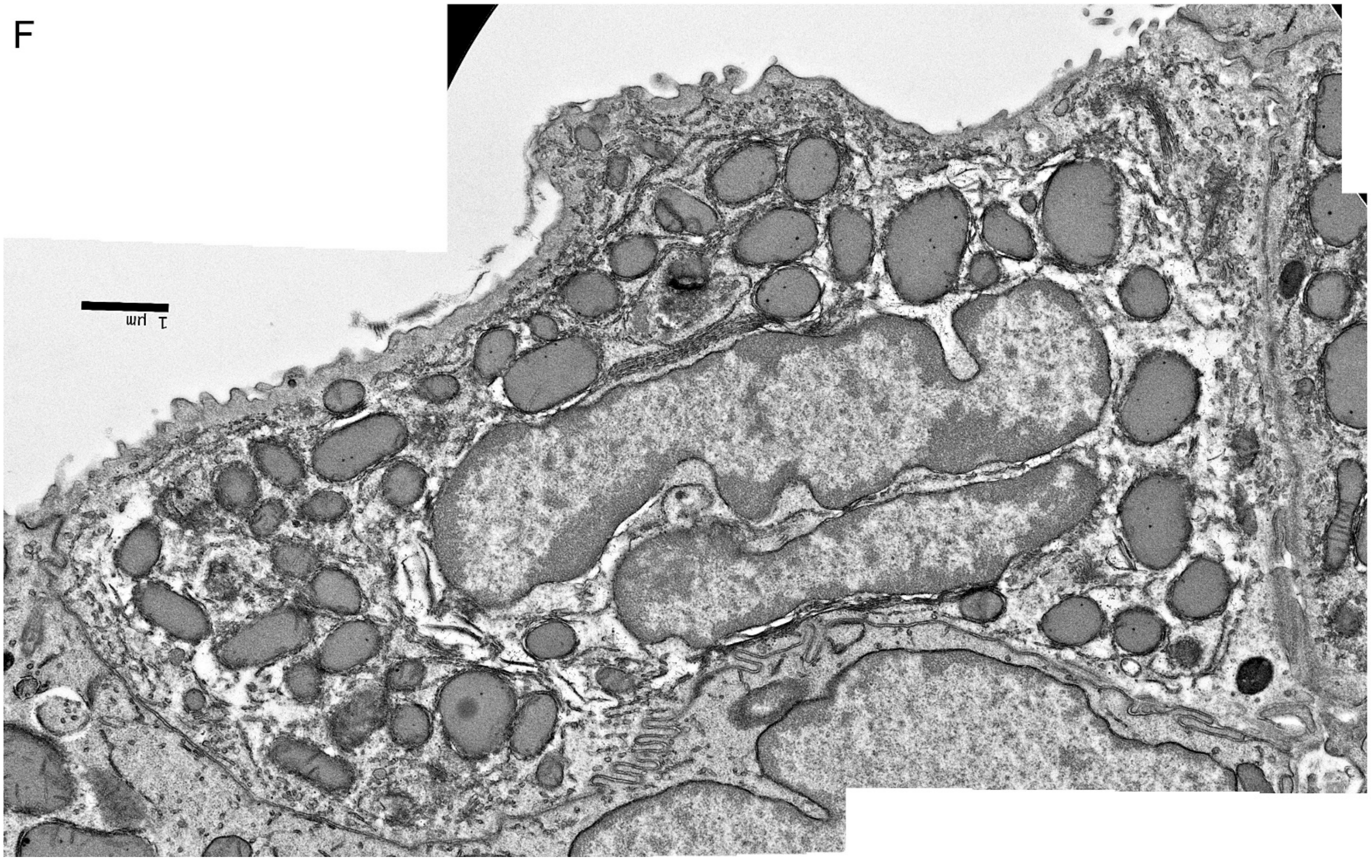

Figure E4

A

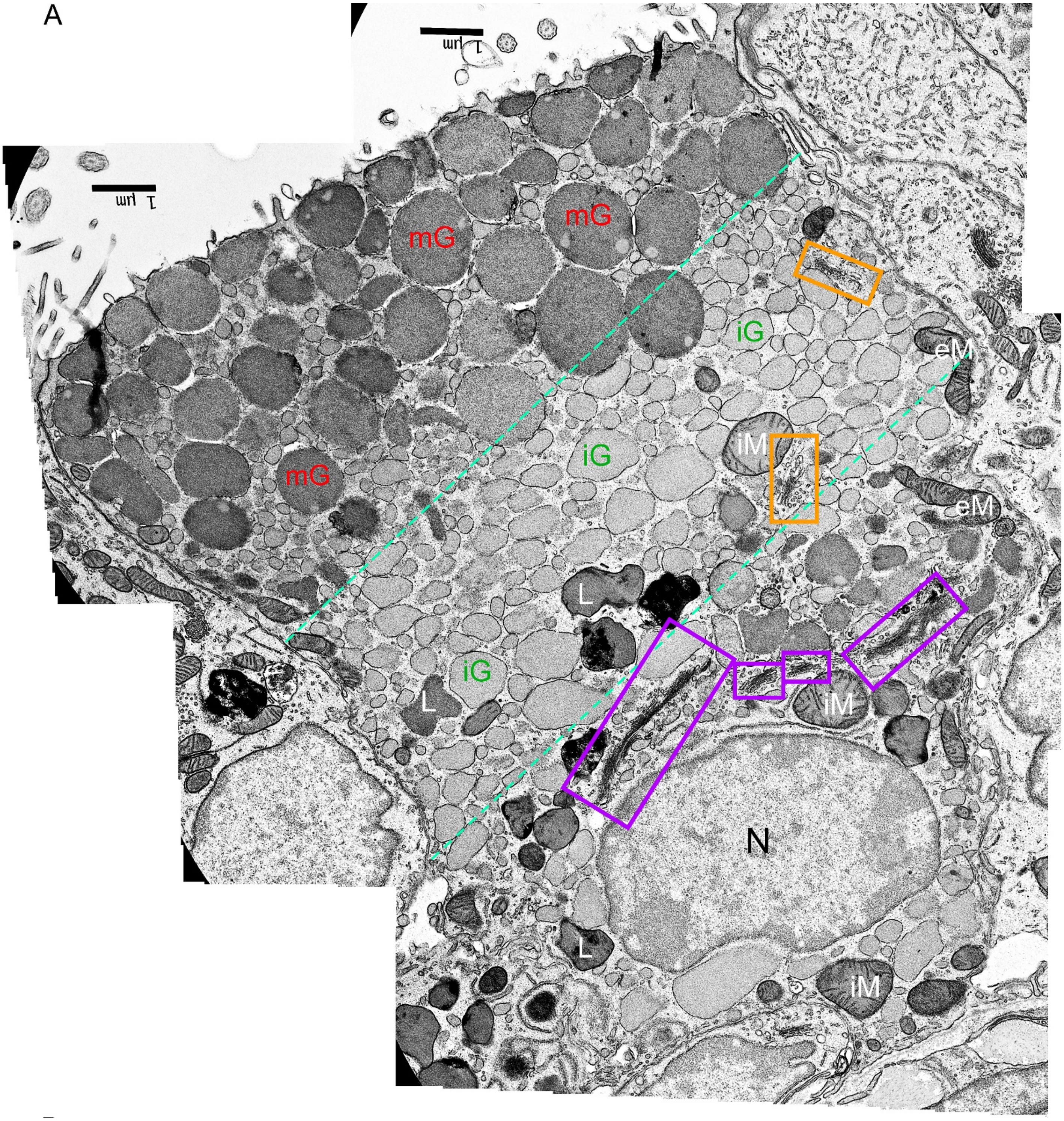

B

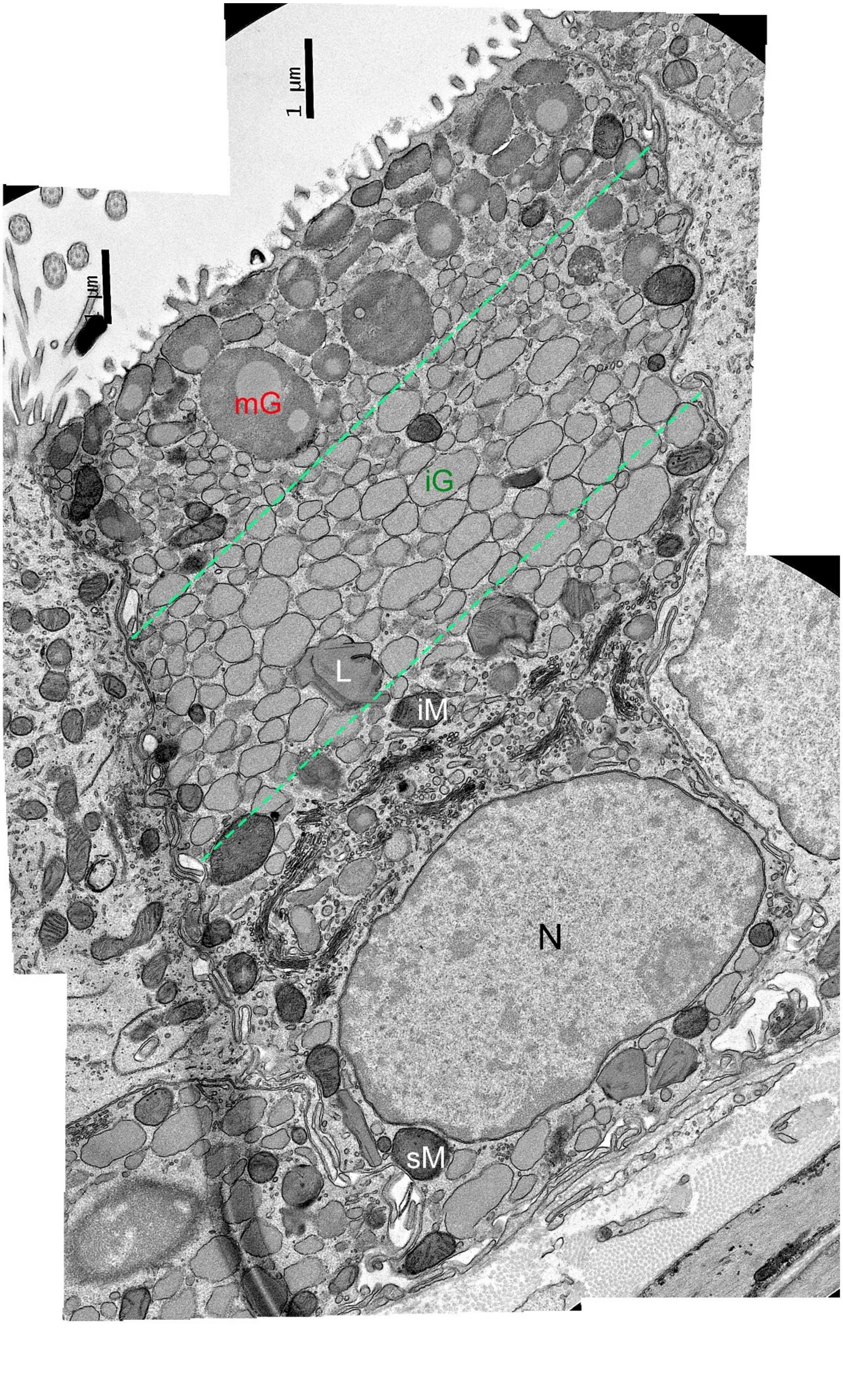

C

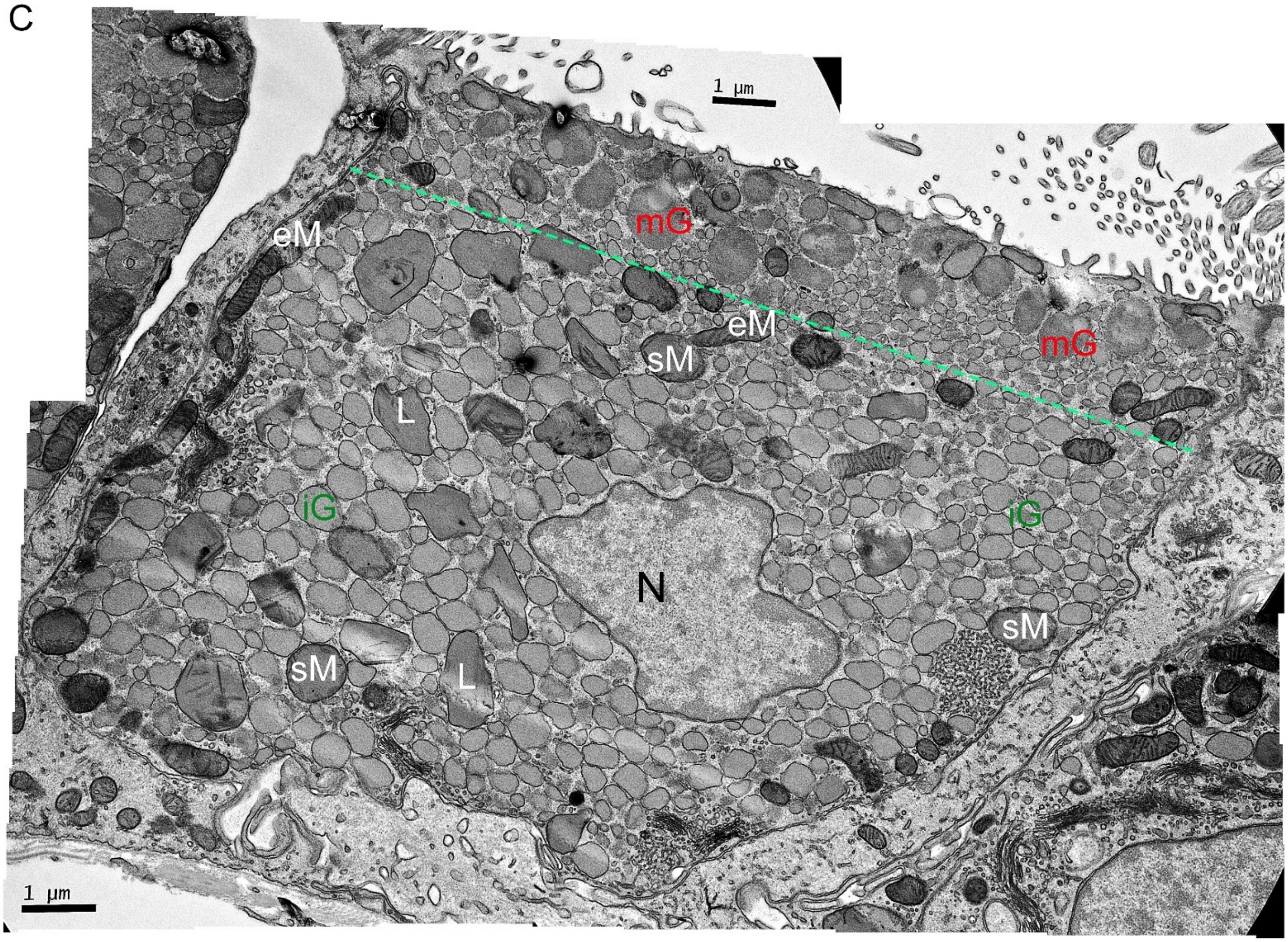

Figure E5

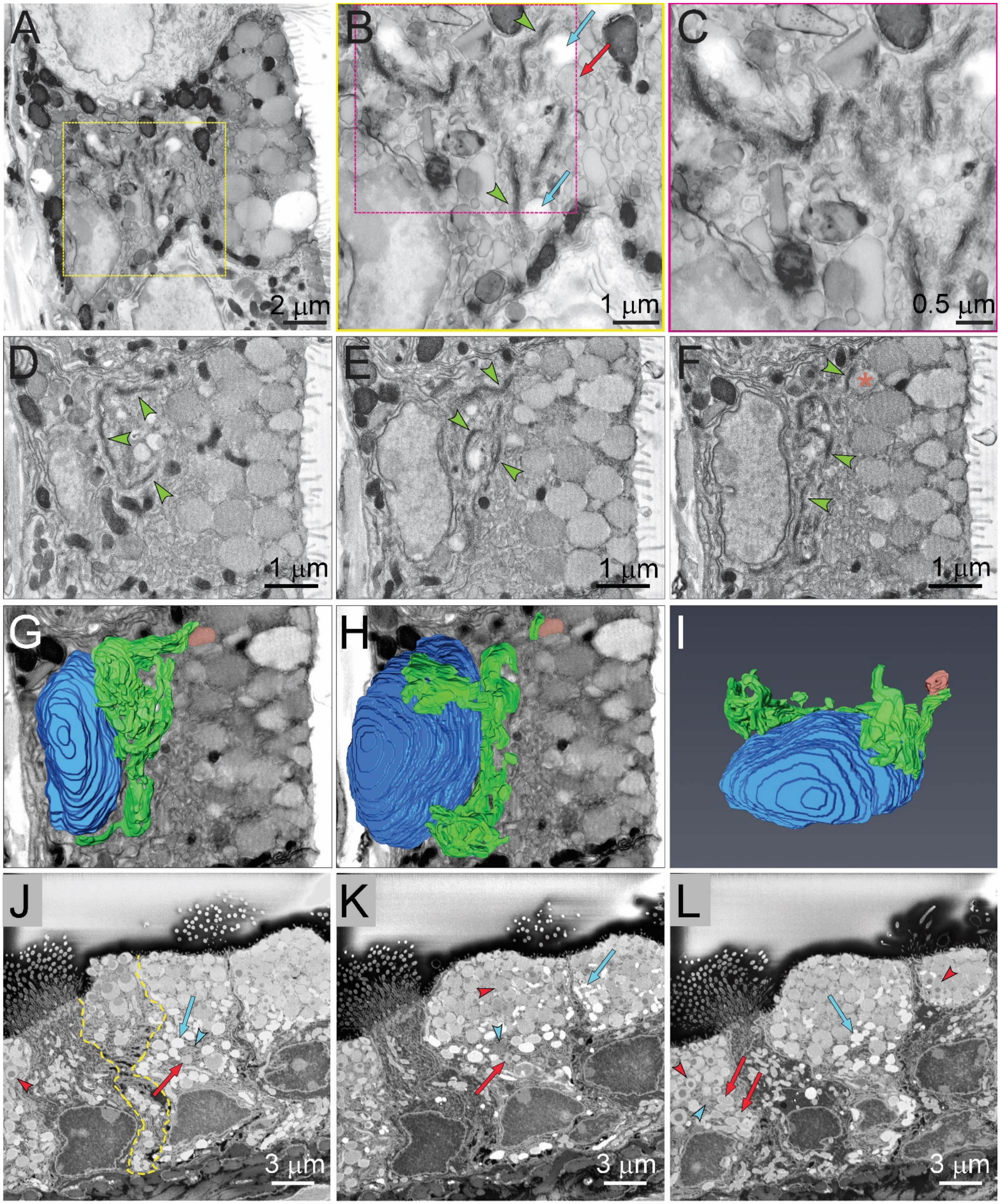

Figure E6

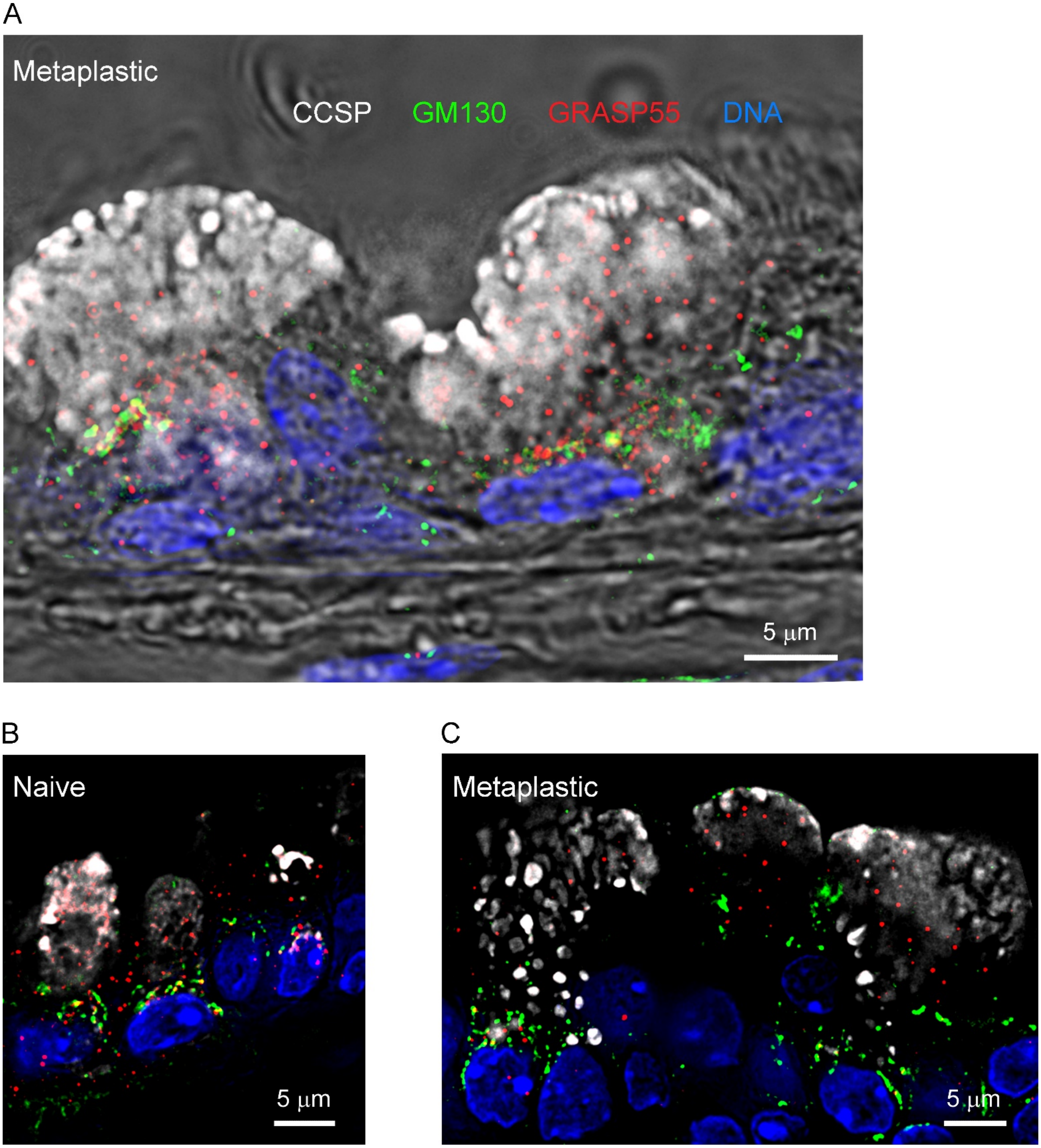
A
Metaplastic
CCSP
GM130
GRASP55
DNA
5 µm
B
Naive
5 µm
C
Metaplastic
5 µm

Figure E7

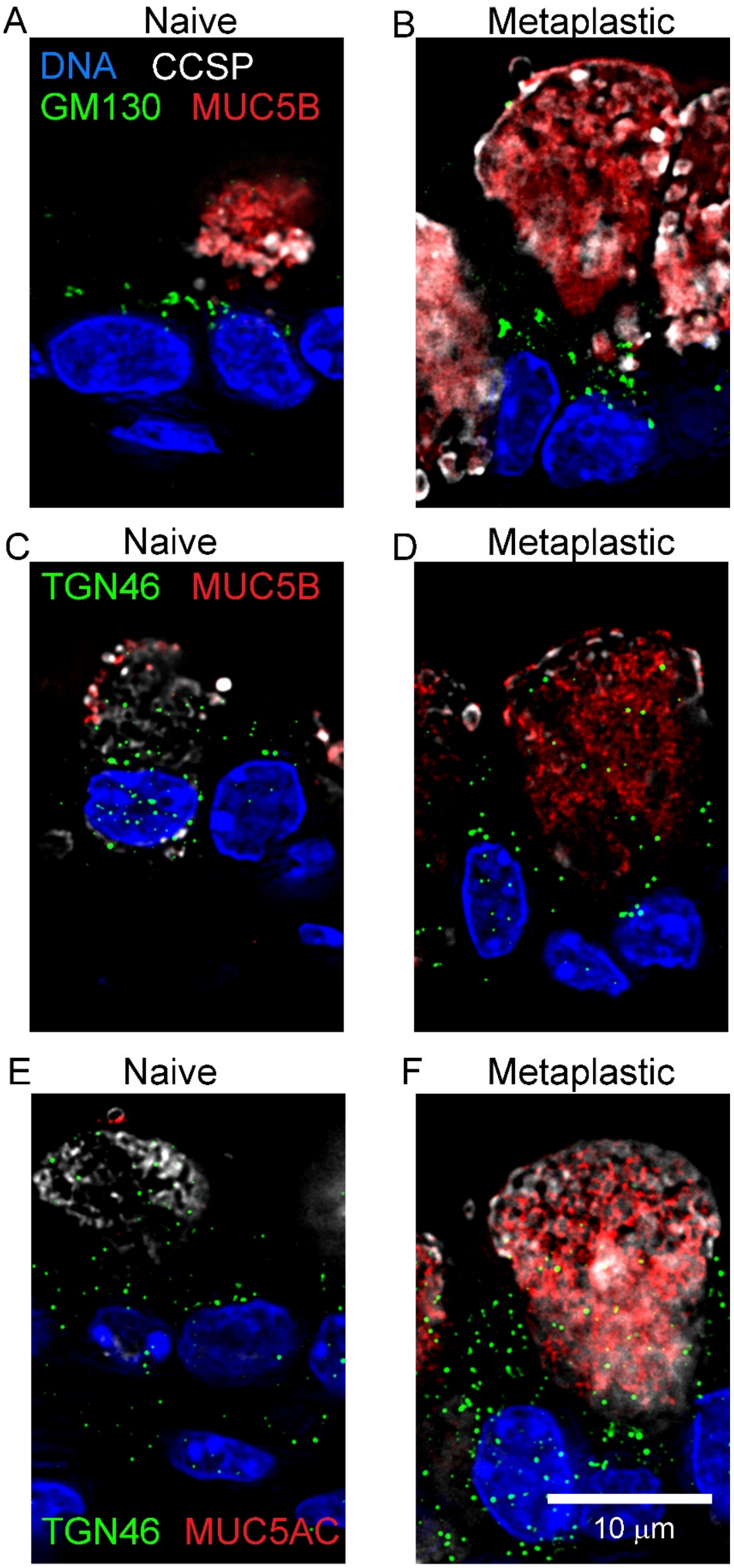

A
Naive
DNA CCSP
GM130 MUC5B
B
Metaplastic
C
Naive
TGN46 MUC5B
D
Metaplastic
E
Naive
TGN46 MUC5AC
F
Metaplastic
10 μm

Figure E8

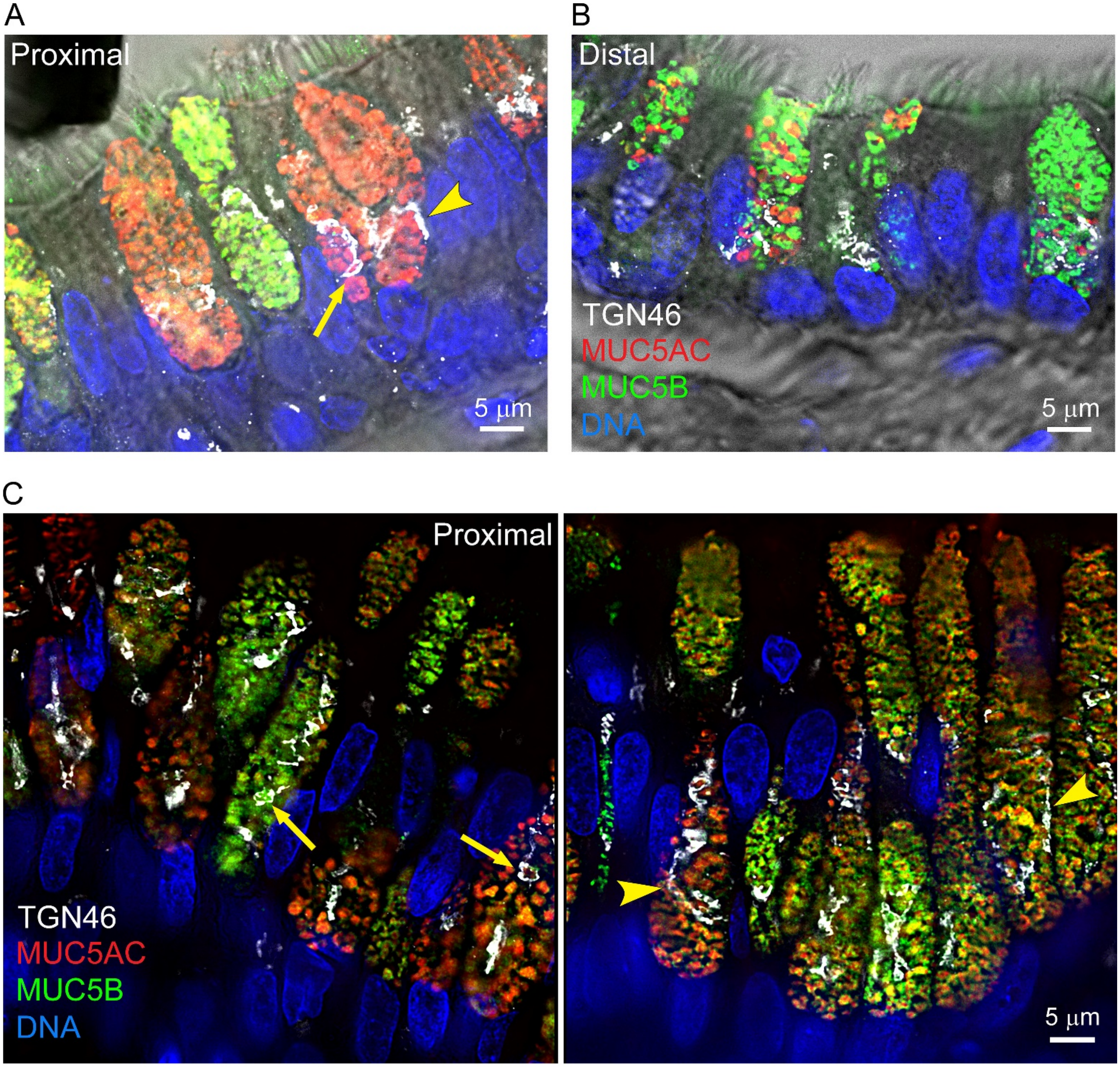
A
Proximal
5 μm
B
Distal
TGN46
MUC5AC
MUC5B
DNA
5 μm
C
Proximal
TGN46
MUC5AC
MUC5B
DNA
5 μm

Figure E9

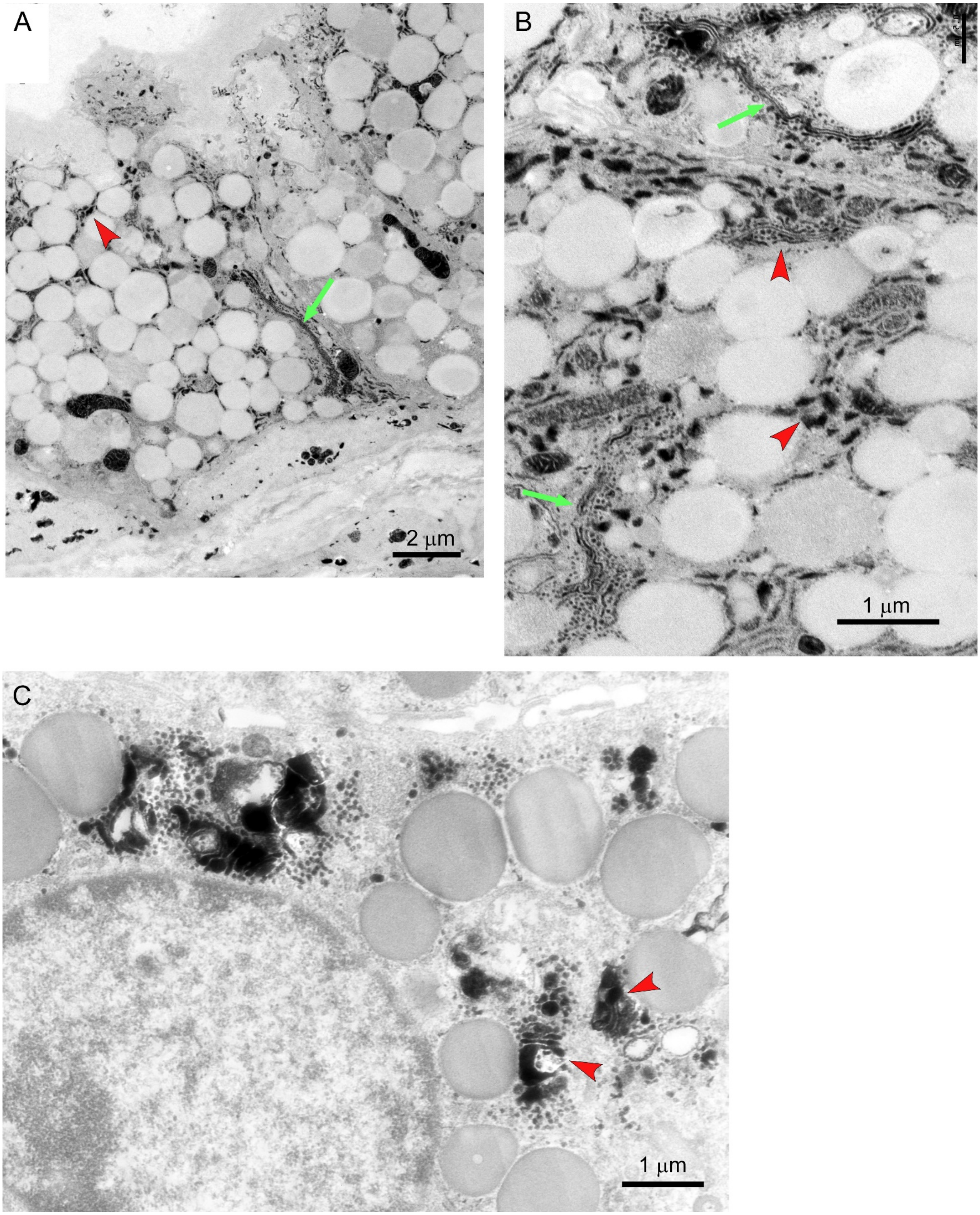

Figure E10

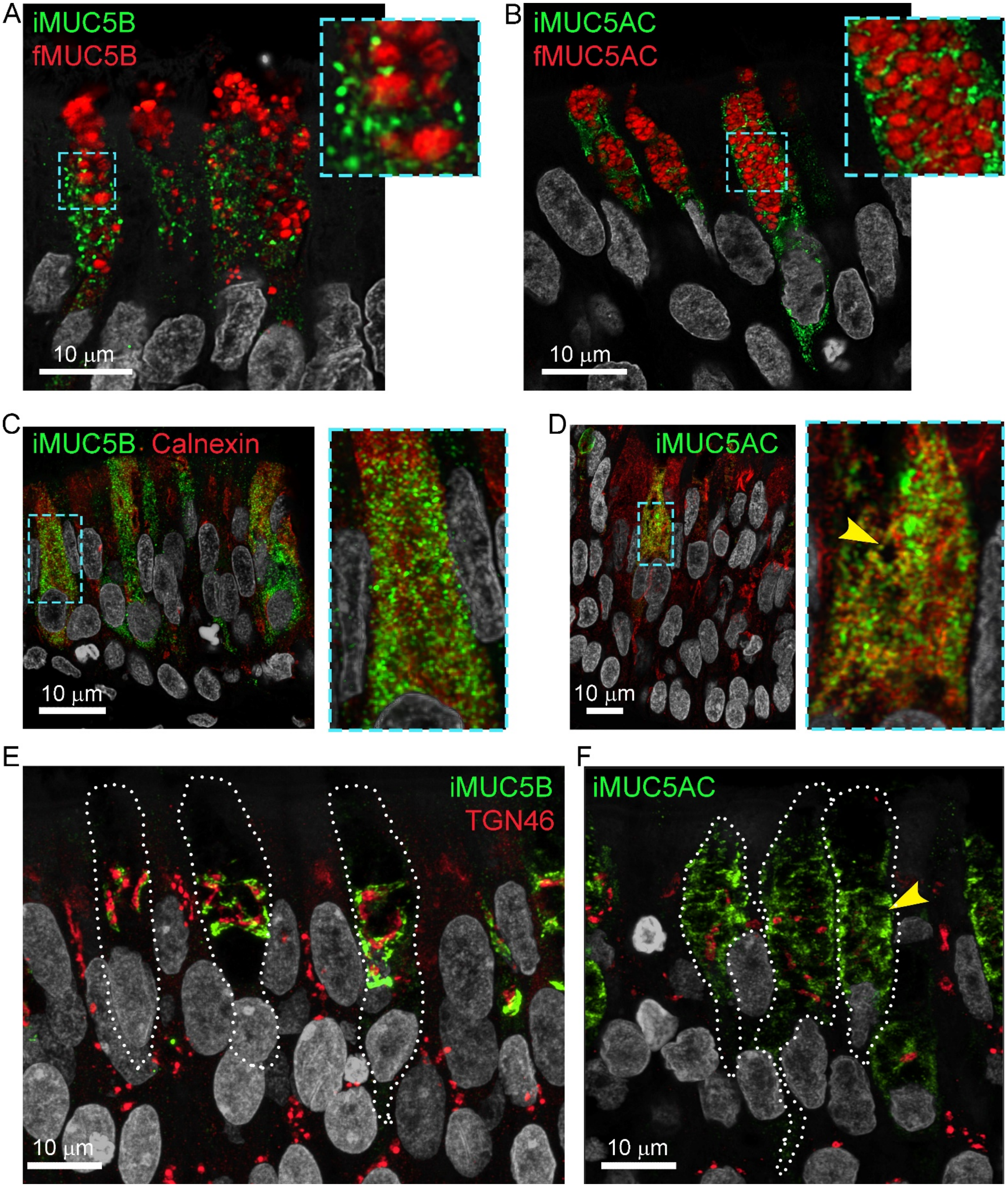

A
iMUC5B
fMUC5B
10 μm
B
iMUC5AC
fMUC5AC
10 μm
C
iMUC5B Calnexin
10 μm
D
iMUC5AC
10 μm
E
iMUC5B
TGN46
10 μm
F
iMUC5AC
10 μm

Figure E11

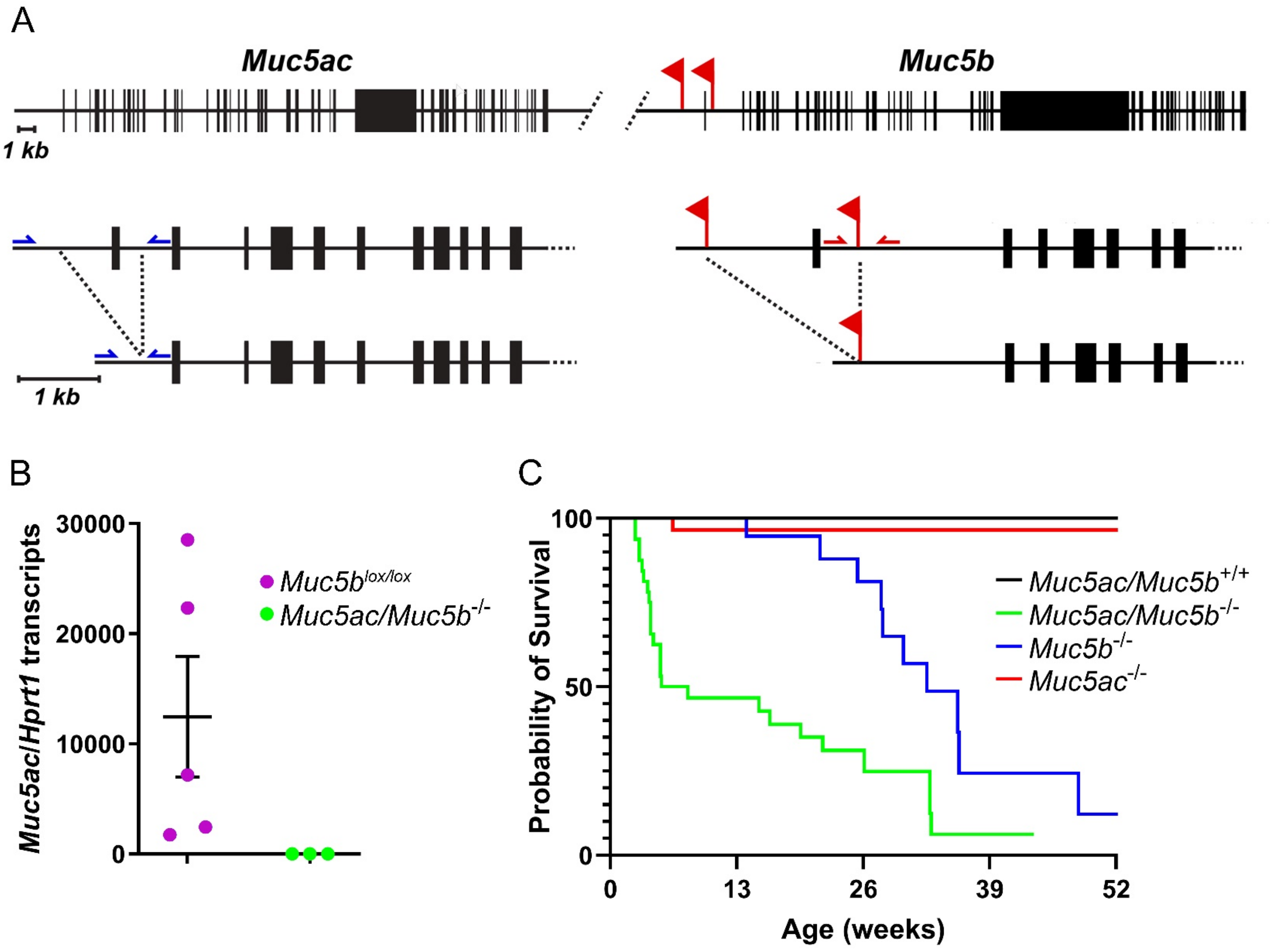

A
Muc5ac
Muc5b
1 kb
1 kb
B
Muc5ac/Hprt1 transcripts
30000
20000
10000
0
Muc5b lox/lox
Muc5ac/Muc5b -/-
C
Probability of Survival
100
50
0
0
13
26
39
52
Age (weeks)
Muc5ac/Muc5b +/+
Muc5ac/Muc5b -/-
Muc5b -/-
Muc5ac -/-
